# Evidence-based treatment of neurogenic orthostatic hypotension and related symptoms

**DOI:** 10.1007/s00702-017-1791-y

**Published:** 2017-10-22

**Authors:** Sabine Eschlböck, Gregor Wenning, Alessandra Fanciulli

**Affiliations:** Department of Neurology, Medical University of Innsbruck, Anichstrasse 35, 6020 Innsbruck, Austria

**Keywords:** Neurogenic orthostatic hypotension, Postprandial hypotension, Syncope, Evidence-based treatment, Grade

## Abstract

Neurogenic orthostatic hypotension, postprandial hypotension and exercise-induced hypotension are common features of cardiovascular autonomic failure. Despite the serious impact on patient’s quality of life, evidence-based guidelines for non-pharmacological and pharmacological management are lacking at present. Here, we provide a systematic review of the literature on therapeutic options for neurogenic orthostatic hypotension and related symptoms with evidence-based recommendations according to the Grading of Recommendations Assessment, Development and Evaluation (GRADE). Patient’s education and non-pharmacological measures remain essential, with strong recommendation for use of abdominal binders. Based on quality of evidence and safety issues, midodrine and droxidopa reach a strong recommendation level for pharmacological treatment of neurogenic orthostatic hypotension. In selected cases, a range of alternative agents can be considered (fludrocortisone, pyridostigmine, yohimbine, atomoxetine, fluoxetine, ergot alkaloids, ephedrine, phenylpropanolamine, octreotide, indomethacin, ibuprofen, caffeine, methylphenidate and desmopressin), though recommendation strength is weak and quality of evidence is low (atomoxetine, octreotide) or very low (fludrocortisone, pyridostigmine, yohimbine, fluoxetine, ergot alkaloids, ephedrine, phenylpropanolamine, indomethacin, ibuprofen, caffeine, methylphenidate and desmopressin). In case of severe postprandial hypotension, acarbose and octreotide are recommended (strong recommendation, moderate level of evidence). Alternatively, voglibose or caffeine, for which a weak recommendation is available, may be useful.

## Introduction

Neurogenic orthostatic hypotension (nOH) is a key feature of cardiovascular autonomic failure, defined by a sustained blood pressure (BP) fall ≥ 20 mmHg systolic and/or ≥ 10 mmHg diastolic within the 3 min of active standing or head-up tilt.

Prevalence of nOH is 30% above 65 years of age (Tilvis et al. 1996) and rises up to 70% in institutionalized patients (Freeman et al. 2011). nOH occurs in up to 80% of patients with multiple system atrophy (MSA) (Ha et al. 2011; Köllensperger et al. 2010; Fanciulli and Wenning 2015), 30% of patients with Parkinson’s disease (PD) (Velseboer et al. 2011) and 100% of patients with pure autonomic failure (PAF) (Goldstein et al. 2015). nOH may also develop in one-third of patients suffering from diabetes mellitus, amyloidosis or spinal cord injury (Low et al. 2004; Wang et al. 2008; Cariga et al. 2002; Illman et al. 2000).

In patients with cardiovascular autonomic failure, a profound and rapid BP fall occurs following food ingestion, due to inability of the sympathetic nervous system to counteract postprandial splanchnic hyperemia, and frequently leads to exacerbation of orthostatic hypotension (Onrot et al. 1985; Mathias et al. 1989, 1991). Post-prandial hypotension, that is a systolic BP drop > 20 mmHg within 2 h from food ingestion, may accompany nOH in up to 50% of patients (Frongillo et al. 1995; Senard et al. 1992).

Symptoms of nOH, including recurrent syncope, dizziness, weakness, nausea, tremulousness, headache or “coat-hanger pain” (pain in the neck and shoulder region) upon standing, may be exacerbated in the early morning, by exercise, heat exposure, dehydration and alcohol consumption. Symptomatic nOH can be severely disabling and poses at higher risk for injurious falls (Rascol et al. 2015). Development of cardiovascular autonomic failure has been also associated with shorter survival, cognitive impairment, as well as higher incidence of cardio- and cerebrovascular events on the long term (Fanciulli et al. 2013).

Management of cardiovascular autonomic failure may result challenging in clinical practice, relying on a combination of non-pharmacological and pharmacological measures, in which the underlying etiology, severity of symptoms and the individual risk–benefit ratio need to be taken into account.

## Aim and methods

In the present paper, we provide an evidence-based overview of non-pharmacological and pharmacological treatment options for nOH and related symptoms of cardiovascular autonomic failure, i.e., post-prandial hypotension and exercise-induced hypotension.

A systematic keyword-based literature search was performed. Articles published in PubMed between January 1985 and March 2017 were screened. Keyword terms comprised: ‘neurogenic orthostatic hypotension’, ‘postural hypotension’, ‘postprandial hypotension’, ‘exercise-induced hypotension’ and ‘syncope’. These terms were searched alone and in combination with ‘non-pharmacological treatment’, ‘pharmacological treatment’, ‘therapy’ and ‘treatment’.

**Fig. 1:**
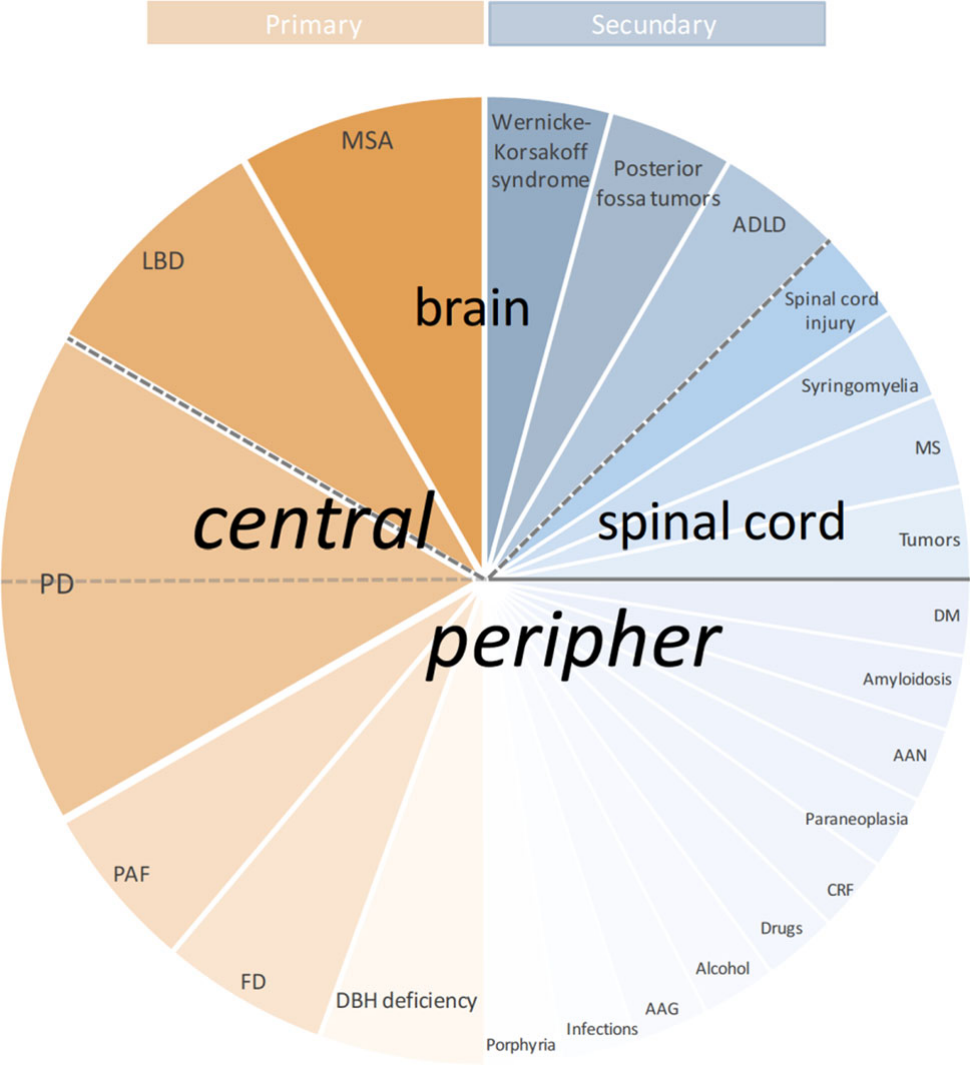
Primary and secondary causes of nOH according to the site of lesion—schematic representation. *AAG* autoimmune autonomic ganglionopathy, *AAN* autoimmune autonomic neuropathy, *ADLD* autosomal dominant leukodystrophy, *CRF* chronic renal failure, *DBH* deficiency dopamine-β-hydroxylase deficiency, *DM* diabetes mellitus, *FD* familial dysautonomia (=hereditary sensory and autonomic neuropathy type III, Riley-Day syndrome), *LBD* Lewy-body dementia, *MS* multiple sclerosis, *MSA* multiple system atrophy, *nOH* neurogenic orthostatic hypotension, *PAF* pure autonomic failure, *PD* Parkinson’s disease
- Allowed study design: randomized controlled trials, open-label trials, case series and case reports.

Following selection criteria were adopted:

- English language.
- Study cohorts: Patients with idiopathic nOH (e.g., pure autonomic failure, multiple system atrophy, Parkinson’s disease) and, with the exception of spinal cord injury, nOH secondary to other diseases [e.g., diabetes mellitus, amyloidosis—for classification of primary and secondary nOH, see also Fig. 1 Primary and secondary causes of nOH according to the site of lesion (Low 2008)].

Additional data were collected by hand-searching references of selected articles according to the abovementioned criteria.

The Grading of Recommendations Assessment, Development and Evaluation (GRADE) approach has been applied for classification of quality of evidence and recommendation level (Leone et al. 2013). The GRADEpro GDT software, provided by the GRADE working group to support grading of quality of evidence and strength of recommendation, has been used for this purpose (“https://gradepro.org”).

## Treatment of nOH and related symptoms: classification of evidence

### Non-pharmacological treatment

#### Effects of water drinking on orthostatic, postprandial and exercise-induced hypotension

The short-term effect of rapid water drinking on orthostatic hypotension has been investigated in studies with sample sizes ranging from 5 to 47 patients with autonomic failure. Nine open-label studies, using different study protocols and outcomes, that investigated the hemodynamic effect of 300–500 ml water have been identified. Six studies reported a significant pressor effect of water drinking that lasted approximately 35 min after ingestion of water (Jordan et al. 1999, 2000; Cariga and Mathias 2001; Shannon et al. 2002; Young and Mathias 2004; Deguchi et al. 2007). In contrast, Mathias et al. 1991 reported that 300 ml water drinking did not have an effect on BP when measured 45 min after water ingestion in patients with MSA and PAF (Mathias et al. 1991). Similar findings were also reported in patients with PD and nOH: ingestion of 500 ml water did not change orthostatic BP when measured 60 min after fluid intake (Senard et al. 1999).

The effect of rapid water drinking on postprandial hypotension has been evaluated in two studies. In seven patients with PAF, rapid water drinking (480 ml, < 5 min before test meal) significantly decreased postprandial BP fall (Shannon et al. 2002). A similar effect was also observed in five patients with MSA (350 ml, < 5 min before test meal) (Deguchi et al. 2007). The effect of water drinking (480 ml distilled water within 5 min) on exercise-induced OH has further been evaluated in a small study in 8 patients with PAF. Although water drinking had an effect on post-exercise OH, no benefit has been demonstrated on BP fall during supine exercise (Humm et al. 2008). Although none of the abovementioned studies reported any adverse event, Jordan et al. 1999 commented that water ingestion may cause supine systolic BP increase more than 100 mm Hg in some patients with underlying cardiovascular autonomic failure (Jordan et al. 1999).

*Quality of evidence* The quality of evidence for water drinking to reduce postural BP drop is very low.

*Safety issues* None.

*Recommendation* The recommendation for water to stabilize BP in patients with autonomic failure is weak.

#### Sodium intake

Few data are available on the effect of salt intake on BP in patients with nOH. Lipp et al. (2005) studied the response to water (500 ml) or to normal saline (500 ml NaCl 0.9%) administered through nasogastric tube in 10 patients with MSA. Results of this single-blinded crossover study demonstrated that ingestion of water increased systolic BP, whereas normal saline hardly showed any pressor response (Lipp et al. 2005).

Raj et al. (2006) evaluated the benefit of water and salt intake in nine patients with nOH of unspecified origin in a randomized, crossover trial. Although both water alone (473 ml) and in combination with salt (2 g NaCl) increased BP, salt intake attenuated global pressor effect (Raj et al. 2006). One further study investigated the effect of water and salt intake in seven patients with MSA: whereas water intake (450 ml) significantly increased BP values, clear soup intake (450 ml soup containing 1.7 g salt) reversed this effect (Z’Graggen et al. 2010).

*Quality of evidence* Based on available data, the quality of evidence for salt intake to reduce orthostatic BP fall is low.

*Safety issues* None.

*Recommendation* The recommendation for increased salt intake to stabilize BP in patients with autonomic failure is weak.

#### Meal size

The intake of smaller meals may improve postprandial hypotension, since food volume and caloric load play an essential role in determining the degree of postprandial splanchnic hyperemia.

The effect of meal size on postprandial hypotension has been studied in one study by Puvi-Rajasingham and Mathias (1996) in seven patients with autonomic failure due to MSA or PAF. Patients were randomized to start either with three meals per day or six meals per day, providing identical daily caloric intake: larger meals resulted in significant lower BP levels in all positions and aggravation of postprandial orthostatic symptoms compared to small meals. However, the mean orthostatic BP fall was unaffected by meal size (Puvi-Rajasingham and Mathias 1996).

*Quality of evidence* The quality of evidence of smaller meals to reduce orthostatic BP drop is very low.

*Safety issues* None.

*Recommendation* Based on limited data, the recommendation for reduction of meal size is weak.

#### Sleeping with head-up tilt

Nocturnal head-up tilt is aimed at reducing nocturnal pressure natriuresis and, therefore, preventing exacerbation of nOH in the early morning.

Two small-sized, open-label studies have been conducted to investigate the effect of nocturnal head-up tilt (12°) on orthostatic BP in patients with nOH. Ten Harkel et al. (1992) reported that nocturnal head-up tilt alone or in combination with fludrocortisone (0.1–0.2 mg) significantly reduced orthostatic BP fall and improved orthostatic symptoms in six patients with nOH. Study period for each intervention was 1 week, including an average follow-up of 14 months (Ten Harkel et al. 1992). Latter findings were replicated by Van Lieshout et al. (1999) in a 3-week open-label study in patients with PAF (*n* = 8): combination of nocturnal head-up sleep and fludrocortisone led to a significant increase in upright BP and minimally improved orthostatic tolerance (van Lieshout et al. 1999). Both studies reported that supine BP remained uninfluenced by treatment. Similar results have been reported in one further patient, where chronic volume expansion due to nocturnal head-up sleep and fludrocortisone administration improved symptoms of orthostatic intolerance (van Lieshout et al. 1991).

*Quality of evidence* Based on limited data, the quality of evidence for nocturnal head-up tilt is very low.

*Safety issues* None.

*Recommendation* The recommendation for sleeping with head-up tilt position during night is weak.

#### Physical countermaneuvers

Physical countermaneuvers may stabilize orthostatic BP through enhancement of peripheral vasoconstriction during orthostatic stress.

The effect of several countermaneuvers has been investigated in a cohort of 17 patients with familial dysautonomia by Tutaj et al. (2006) in an open-label, randomized crossover trial: bending forward, squatting and abdominal compression (20 mmHg), but not leg crossing, significantly ameliorated orthostatic BP fall in this cohort (Tutaj et al. 2006).

In contrast, leg crossing, but not tiptoeing, was able to combat orthostatic BP fall in patients with nOH due to PAF (Ten Harkel et al. 1994). Two further open-label studies and one case report are available confirming that a range of physical countermaneuvers attenuate nOH in chronic autonomic failure (Bouvette et al. 1996; van Lieshout et al. 1992; Wieling et al. 1993). In addition, Smit et al. (2004) evaluated the combination of legs crossing and abdominal compression in an open-label, randomized study. The combination of leg crossing and abdominal compression (20–40 mmHg) was more effective than abdominal compression alone in stabilizing orthostatic BP. Leg crossing alone also improved standing BP in 12 patients with nOH due to PAF, MSA and diabetes mellitus (Smit et al. 2004).

*Quality of evidence* The quality of evidence of physical countermaneuvers to reduce orthostatic BP drop in patients with autonomic failure is very low.

*Safety issues* None.

*Recommendation* Based on available data, there is a weak recommendation for use of physical countermaneuvers.

#### Abdominal binders and compression stockings

The efficacy of compression garments applied to different parts of the body to reduce venous pooling has been assessed in several small-sized studies.

One randomized, single-blind, placebo-controlled, crossover trial in 15 patients with PD and nOH by Fanciulli et al. (2016a) showed that abdominal binders, exerting a compression of 20 mmHg, significantly diminished BP fall upon head-up tilt without influencing supine BP values. In a 4-week open-label follow-up, daily use of abdominal binders also significantly improved nOH-related disability, assessed by means of the Orthostatic Hypotension Questionnaire (OHQ) (Fanciulli et al. 2016a).

One further randomized, crossover trial using an open-label study design has been conducted to evaluate the effect of abdominal compression with conventional and adjustable binders in 13 patients with nOH by Figueroa et al. (2015): mild abdominal compression (10 mmHg) prior to rising proved to ameliorate nOH. Although an increase in abdominal compression upon standing up to maximal tolerance did not further ameliorate nOH, decompression tended to aggravate orthostatic BP fall (Figueroa et al. 2015). The benefit of abdominal compression has been also reported by Smit et al. (2004) and Tutaj et al. (2006) using an abdominal compression that ranged from 20 to 40 mmHg (see description above).

Studies that systematically evaluated the effect of compression stockings are limited. Denq et al. (1997) showed that either contemporary compression of calves, thighs and low abdomen (40 mmHg each) or of the abdomen alone significantly improved nOH 14 patients with MSA, PAF and diabetic neuropathy. In contrast, the sole leg compression (calves and/or thighs) was less effective (Denq et al. 1997). In a young patient with insulin-dependent diabetes mellitus and nOH, symptomatic relief was reported with use of a G-suit (Elizondo et al. 1996).

*Quality of evidence* The quality of evidence for abdominal binders to reduce orthostatic BP fall is moderate, for compression stockings is very low.

*Safety issues* None.

*Recommendation* The recommendation for the use of abdominal binders to improve orthostatic BP fall in patients with autonomic failure is strong, for compression stockings is weak.

### Pharmacological treatment

#### Orthostatic hypotension

##### Midodrine

Midodrine, a peripheral α_1_-selective adrenoceptor agonist and prodrug of the active metabolite desglymidodrine, increases blood pressure by inducing vasoconstriction (Pittner et al. 1976).

Three randomized double-blind trials, with either cross-over study design (Wright et al. 1998) or parallel group (Jankovic et al. 1993; Low et al. 1997), that directly compared the effect of midodrine in a dosage of 2.5–30 mg per day versus placebo, have been conducted. The potent pressor effect of midodrine on standing BP was shown in all three studies with sample sizes ranging from 25 to 171 patients with autonomic failure and a study period (randomized, placebo-controlled phase) lasting up to 4 weeks.

Previously, Kaufmann et al. (1988) had investigated midodrine (25–40 mg/day) in combination with fludrocortisone (0.1 mg) or fludrocortisone alone in a randomized controlled trial with seven patients with MSA and PAF, demonstrating that both combination therapy and fludrocortisone alone were effective in only half of the patients. In the non-responder group, midodrine and fludrocortisone even decreased upright BP with respect to placebo.

Two further randomized, placebo-controlled, comparative studies with a cross-over design are available. Fouad-Tarazi et al. (1995) demonstrated that midodrine administered in a dosage of 2.5–10 mg three times a day, but not ephedrine (6–24 mg/day), significantly improved standing BP and the ability to stand in eight patients with MSA and PAF.

More recently, midodrine (5–10 mg/day) was compared to atomoxetine (18 mg/day) in 69 patients with nOH due to PAF, MSA or PD by Ramirez et al. (2014). Although both treatment arms significantly increased upright BP with respect to placebo, atomoxetine led to a higher increase of standing BP than midodrine. Nonetheless, the lack of a wash-out phase limits interpretation of this study, given that plasmatic half-live of atomoxetine may range from 5 h (extensive metabolizers) to 21 h (poor metabolizers) (Sauer et al. 2005).

The effect of midodrine on nOH has been investigated in further small-sized, non-randomized, open-label studies in patients with autonomic failure of different etiologies [familial dysautonomia (Axelrod et al. 1995), idiopathic OH (Axenti et al. 1993) and PAF/autonomic neuropathy (Schrage et al. 2004)] supporting efficacy of midodrine for treatment of nOH.

*Quality of evidence* The quality of evidence of midodrine to reduce orthostatic BP fall in patients with autonomic failure is high.

*Safety issues* The main safety concern of midodrine is supine hypertension. Further side effects include urinary retention and uncomfortable reactions such as paresthesia, piloerection, chills, scalp and generalized pruritus.

*Recommendation* Based on available data, there is a strong recommendation for use midodrine for treatment of nOH. Monitoring for exacerbation of supine hypertension and increase of post-void residual urine volume is required.

##### Droxidopa

The efficacy of droxidopa (l-Dihydroxyphenylserine, l-DOPS), a precursor of norepinephrine, has been investigated in five randomized controlled trials involving more than 600 patients with nOH mainly due to PAF, MSA and PD, in a dosage ranging from 200 to 1800 mg, for a placebo-controlled, randomized period of up to 8 weeks.

Whereas four clinical trials demonstrated a significant difference in diverse outcomes favouring droxidopa over placebo [change in supine and upright systolic and diastolic BP (Freeman et al. 1999); supine and standing BP, HR, orthostatic tolerance (Kaufmann et al. 2003); change in OHQ composite score (Kaufmann et al. 2014); change in item 1 (dizziness/lightheadedness) of the Orthostatic Hypotension Symptoms Assessment (OHSA) scale (Hauser et al. 2015)], two further studies failed to meet the primary endpoint, defined as change in OHSA item 1 (dizziness/lightheadedness) (Biaggioni et al. 2015) and change in OHQ composite score (Hauser et al. 2014).

In details, Biaggioni et al. (2015) administered droxidopa, 100–600 mg three times a day, in 101 patients with autonomic failure. The primary efficacy end-point, defined as mean change of OHSA item 1 from randomization to study end, did not significantly differ from placebo (Biaggioni et al. 2015). Nonetheless, a conducted post hoc analysis showed a significant effect of droxidopa measured by OHQ composite score (Biaggioni et al. 2015). The possibility of a substantial carryover effect, explaining the failure of droxidopa in primary outcome, has been discussed, since patients randomized to placebo reported a sustained relief of the orthostatic dizziness/lightheadedness score and standing systolic BP at the end of the study. Besides, symptomatic improvement simply related to study participation has been hypothesized, possibly due to better adherence to non-pharmacological countermeasures during observation period (Kaufmann et al. 2015).

A pre-planned interim efficacy analysis of study 306 A in 51 patients with PD failed to show a significant difference for droxidopa with respect to placebo in primary endpoint, which was defined as change in OHQ composite score after 8 weeks (Hauser et al. 2014). Primary outcome was, therefore, modified to changes in item 1 of the OHSA score at 1-week-treatment for patients subsequently enrolled in study 306 B. Pooled results from study 306A and B demonstrated significant improvement in the dizziness/lightheadedness OHSA score (item 1) after 1-week-treatment in 171 patients with PD (Hauser et al. 2015).

Several additional small-sized studies (Carvalho et al. 1997; Mathias et al. 2001; Kaufmann et al. 1991; Matsubara et al. 1990; Goldstein and Sharabi 2009) and case reports (Sakoda et al. 1958; Kachi et al. 1988; Man in’t Veld et al. 1987; Biaggioni and Robertson 1987) further report benefit from droxidopa in patients with nOH due to MSA, PAF, PD, familial amyloidotic polyneuropathy and dopamine-beta-hydroxylase deficiency.

*Quality of evidence* The quality of evidence of droxidopa to reduce nOH-related symptoms is moderate based on missing long-term data.

*Safety issues* Possible side effects include hypertension, headache, dizziness and nausea. Falls, urinary tract infections, headache, syncope and dizziness have been reported at open-label follow-up, though systematic post-marketing studies to define rate of adverse events in clinical practice are not available yet (Kaufmann et al. 2015).

*Recommendation* recommendation for droxidopa to treat orthostatic intolerance in patients with cardiovascular autonomic failure is strong. Regular BP monitoring is required, since treatment may exacerbate supine hypertension.

##### Fludrocortisone

Fludrocortisone, a synthetic mineralocorticoid, increases BP by expanding intravascular volume through sodium and water retention.

Evidence-based data on fludrocortisone for treatment of nOH are limited. Only two randomized, double-blind, crossover studies are available.

Kaufmann et al. (1988) investigated the effect of fludrocortisone in a dosage of 0.1 mg per day in combination with placebo or midodrine (25–40 mg/day) in 7 patients with nOH due to MSA or PAF. Fludrocortisone, administered for a period of 1 week, increased upright BP only in half of patients. The remaining patients did not respond to medication and upright BP even decreased (Kaufmann et al. 1988).

Schoffer et al. (2007) compared the efficacy of fludrocortisone and domperidone in a randomized, double-blind comparative study. In 17 patients it was shown that both fludrocortisone (0.1 mg/day) as well domperidone (30 mg/d) improved primary outcomes measured by Composite Autonomic Symptom Scale (COMPASS) and clinical global impression of change (CGI) within 3 weeks of treatment. In addition, fludrocortisone and domperidone showed a trend, though not significant, towards reduction of BP fall upon tilt-table testing (Schoffer et al. 2007).

The anti-hypotensive effect of fludrocortisone has been further demonstrated in small-sized, open-label studies in patients with MSA, PD, PAF and hypoadrenergic OH). (Ten Harkel et al. 1992; van Lieshout et al. 1991, 1999; Matsubara et al. 1990; Hakamäki et al. 1998).

*Quality of evidence* The quality of evidence of fludrocortisone to reduce orthostatic BP fall is very low.

*Safety issues* Main side effect that has been reported is nocturnal hypertension (Hakamäki et al. 1998). Further adverse events include headache, nausea, dizziness as well as generalized or localized edema and hypokalemia (Ricci et al. 2015). Data for long-term administration are limited. Experimental data suggest that long-term use is associated with end-organ damage (Norcliffe-Kaufmann et al. 2013) and increased risk of renal and cardiac fibrosis (Kaufmann 2016, personal communication).

*Recommendation* Recommendation for use of fludrocortisone for treatment of nOH is weak, based on quality of evidence and safety issues. Monitoring of supine BP and electrolytes is required.

##### Pyridostigmine

The therapeutic rationale of the cholinesterase-inhibitor pyridostigmine for treatment of nOH relies on enhancement of cholinergic sympathetic ganglionic transmission, secondarily inducing BP rise. Such effect is especially to be expected during sympathetic activation, i.e., when subjects are in the upright position (Biaggioni 2014).

Based on two prospective open-label trials (Singer et al. 2003, 2006a), Singer et al. (2006b) conducted a double-blind, randomized, 4-arms (pyridostigmine 60 mg alone, placebo, pyridostigmine 60 mg in combination with 2.5 or 5 mg midodrine), cross-over study. In 58 patients with nOH (MSA, PAF, diabetic and autoimmune autonomic neuropathy), the authors demonstrated that pyridostigmine significantly improved standing diastolic BP one hour after administration. Importantly, no aggravation of supine hypertension was observed (Singer et al. 2006b). In a follow-up, open-label study pyridostigmine maintained favorable effects, especially in patients with less severe nOH without major side effects (Sandroni et al. 2005).

Shibao et al. (2010) evaluated the effect of pyridostigmine (60 mg) versus yohimbine (5.4 mg) in 31 patients with autonomic failure (MSA, PAF, PD) in a single-blind, randomized, controlled trial. In contrast to Singer et al. (2006b), this study could neither confirm that pyridostigmine increases standing diastolic BP 1 h after drug administration, nor that it improves pre-syncopal symptoms. The authors commented that the study population was more severely affected than in previous studies, partially explaining the conflicting results (Shibao et al. 2010).

*Quality of evidence* Very low, due to inconsistency of results.

*Safety issues* Side effects include gastrointestinal symptoms and urinary urgency (Biaggioni 2014).

*Recommendation* The recommendation for use of pyridostigmine to improve nOH is weak.

##### Yohimbine

Yohimbine, an α2-adrenergic antagonist, leads to augmentation of sympathetic activity at CNS level, thus increasing norepinephrine release from adrenergic nerve terminals (Biaggioni et al. 1994a).

For yohimbine, three small-sized randomized controlled trials in up to 31 patients with nOH are available, showing inconsistent results.

Shibao et al. (2010) investigated the effect of yohimbine and pyridostigmine on standing diastolic BP one hour after administration in 31 patients with nOH due to MSA, PAF or PD. Only yohimbine (5.4 mg) significantly increased standing diastolic BP, seated systolic and diastolic BP and significantly improved nOH-related symptoms (Shibao et al. 2010). The positive pressor effect of yohimbine has been further reported in small-sized studies (*n* = 8–35) (Jordan et al. 1998, 2000; Onrot et al. 1987) in patients with autonomic failure.

In contrast to abovementioned findings, Senard et al. (1993) did not observe any BP increase at 24-h ambulatory BP monitoring in 17 patients with PD after 4-week treatment with yohimbine (2 mg three times a day) (Senard et al. 1993).

Similarly, Okamoto et al. (2012) reported negative results in a single-blind, crossover study with 17 patients with nOH due to PAF and PD: neither yohimbine (5.4 mg), nor atomoxetine (18 mg) alone significantly increased seated systolic BP 1 h after drug administration or improved orthostatic symptoms. Only co-administration of both yohimbine and atomoxetine increased seated systolic BP and orthostatic tolerance (Okamoto et al. 2012).

Limits of abovementioned RCTs include lack of a wash-out phase and/or measurement of supine BP.

*Quality of evidence* The quality of evidence for yohimbine to increase BP and orthostatic tolerance is very low.

*Safety issues* No side effects reported, effect on supine BP unknown.

*Recommendation* The recommendation for use of yohimbine is weak, due to very low and inconsistent quality of evidence.

##### Atomoxetine

Atomoxetine, which augments norepinephrine concentration in the synaptic gap by selectively blocking norepinephrine transporter, has been evaluated in three randomized, placebo-controlled trials, with a sample size ranging from 21 to 69 subjects.

As mentioned above, Okamoto et al. 2012 showed that neither atomoxetine (18 mg) nor yohimbine (5.4 mg) significantly changed seated systolic BP or orthostatic tolerance 1 h post-treatment in a cohort of 17 patients with nOH due to PAF and PD. Only the combination of both increased seated systolic BP and improved nOH-related symptoms (Okamoto et al. 2012).

In contrast, Shibao et al. (2007a) demonstrated that atomoxetine (18 mg) significantly increases seated and standing systolic BP in patients with central autonomic failure (MSA), but not in patients with peripheral autonomic failure (PAF/PD) (Shibao et al. 2007a).

Ramirez et al. (2014) further reported that both atomoxetine (18 mg, single dose) and midodrine (5–10 mg, single dose) significantly increased seated and upright BP with respect to placebo in a randomized, single-blind, crossover trial with 65 patients with nOH due to MSA, PAF, PD or of undetermined origin. Atomoxetine showed a higher increase in standing systolic BP with respect to midodrine. Atomoxetine, but not midodrine, also improved nOH-related symptoms with respect to placebo (Ramirez et al. 2014).

A possible carry-over effect cannot, however, be excluded in above mentioned studies, since washout-phases were not described in detail. The effect of atomoxetine on supine BP was not mentioned.

*Quality of evidence* Based on available literature, the quality of evidence for atomoxetine to raise standing BP is low.

*Safety issues* No side effects reported, effect on supine BP remains unclear.

*Recommendation* The recommendation for atomoxetine for treatment of nOH is weak, based on quality of evidence and pending safety issues.

##### Fluoxetine

The rationale for use of fluoxetine, a selective serotonin reuptake inhibitor, to improve nOH is unknown. One small, open-label study in five patients with nOH of varying etiologies (PAF, renal failure, diabetes mellitus) revealed a positive impact of fluoxetine (20 mg once daily) on nOH after 6–8 weeks of treatment (Grubb et al. 1994). In addition, in an open-label study, Montastruc et al. (1998) demonstrated that chronic administration of fluoxetine (20 mg; 1-month treatment) significantly reduced orthostatic systolic BP fall and provided symptomatic relief in 14 patients with PD (Montastruc et al. 1998).

*Quality of evidence* The quality of evidence for fluoxetine to improve nOH is very low.

*Safety issues* Common side effects are gastrointestinal complaints, headache and dizziness (Wernicke 2004).

*Recommendation* Based on the quality of evidence, the recommendation for fluoxetine for treatment of nOH is weak.

##### Ergot alkaloids

Ergotamine, an ergot alkaloid, and its derivate dihydroergotamine increase BP through α-adrenergic vasoconstriction of arteries and veins (Perrin 1985).

Ergotamine has been investigated in a small-sized (*n* = 8), placebo-controlled, crossover study. Biaggioni et al. 1990 reported that inhalation of ergotamine (1 puff, 0.36 mg) proved effective compared to placebo in increasing seated and upright BP, as well as prolonging standing time before onset of nOH-related symptoms (Biaggioni et al. 1990). It is, however, unclear whether patients were randomly assigned to begin either with treatment or placebo and if the study included a wash-out phase. In one additional case report (Siminoski et al. 1988) aerosol administration of ergotamine has been described in a patient with multi-domain autonomic failure.

Dihydroergotamine, in a dosage ranging from 6.5 to 13 μg/kg, was investigated in two small-sized, randomized, placebo-controlled studies with a crossover design (Hoeldtke et al. 1989b; Hoeldtke and Israel 1989), in which an increase of upright BP was shown. In both studies, main limitation was represented by the lack or rather unclear description of a wash-out phase.

In addition, one open-label study (Victor and Talman 2002) compared intravenous administration of ergotamine (0.15 mg) versus oral clonidine (0.4 mg) in four patients with severe autonomic failure: whereas both drugs increased supine BP, dihydroergotamine was more effective in contrasting orthostatic BP fall.

Quality of evidence: Based on limited data, the quality of evidence for both ergotamine and dihydroergotamine to reduce orthostatic BP fall in patients with autonomic failure is very low.

Safety issues: common side effects of ergot alkaloids are nausea, vomiting, paresthesias and fatigue. Severe adverse effects include fibrosis (retroperitoneal, cardiac, pleural, pulmonary), peripheral vasoconstriction and ergotism (Schiff 2006).

Recommendation: in consideration of quality of evidence and safety issues, the recommendation for ergotamine and dihydroergotamine for treatment of nOH is weak.

##### Recombinant erythropoietin

Erythropoietin is reckoned to combat nOH by stimulating red cell mass production, thus increasing circulating blood volume and tissue oxygenation.

No randomized controlled trials are available for the treatment of nOH with erythropoietin in the setting of autonomic failure. Several small-sized (sample size up to 24 patients), open-label studies and case reports in patients with primary autonomic failure or nOH secondary to diabetes mellitus type 1 or familial amyloidosis reported a beneficial effect of erythropoietin on nOH (Hoeldtke and Streeten 1993; Perera et al. 1995; Biaggioni et al. 1994b; Winkler et al. 2001; Beirão et al. 2008; Kawakami et al. 2003).

*Quality of evidence* The quality of evidence for erythropoietin to increase orthostatic BP in patients with autonomic failure is very low.

*Safety issues* Adverse events include flu-like symptoms, allergic reactions, hypertension and increased risk of thrombosis, among others (Eagleton and Littlewood 2003).

*Recommendation* The recommendation for erythropoietin to treat nOH in patients with autonomic failure is weak.

##### Ephedrine

Available evidence for ephedrine, a nonspecific direct and indirect α- and β-adrenoceptor agonist (Fouad-Tarazi et al. 1995), is scarce.

Efficacy and safety of ephedrine have been compared to midodrine in a randomized, double-blind, placebo-controlled, crossover study in eight patients with PAF and MSA by Fouad-Tarazi et al. (1995): ephedrine neither improved standing BP, nor the ability to stand. In contrast, ephedrine significantly increased supine systolic and diastolic BP (Fouad-Tarazi et al. 1995).

*Quality of evidence* Based on limited data, the quality of evidence for ephedrine to improve standing BP is very low.

*Safety issues* Adverse events include exacerbation of supine hypertension, dizziness, lightheadedness, photosensitivity and loss of balance.

*Recommendation* In consideration of quality of evidence, efficacy and safety profile, the recommendation for ephedrine to treat orthostatic hypotension in patients with autonomic failure is weak.

##### Other ephedra alkaloids

Phenylpropanolamine was investigated in an open study in 14 patients with nOH of varying etiologies (PAF, MSA, diabetes mellitus). Administration of 12.5 to 25 mg phenylpropanolamine led to a significant increase in seated BP in patients with autonomic failure (Biaggioni et al. 1987).

Jordan et al. (1998) used a single-blinded, placebo-controlled design to administer various pressor agents, including phenylpropanolamine. Phenylpropanolamine in a dosage of 12.5 to 25 mg elicited a significant pressor effect with respect to placebo. Notwithstanding, this study was not primarily designed for therapeutic purposes and presents several limitations: randomization is not mentioned; due to individual contraindications, not every patient received each study medication and no clear description of wash-out phases is provided. The authors further state that the study was carried over a period of several years (Jordan et al. 1998).

Jordan et al. (2004) confirmed in an open-label study in 13 patients with autonomic failure that phenylpropanolamine (12.5 and 25 mg) and pseudoephedrine (a stereoisomer of ephedrine, 30 mg per os) significantly increased BP. The pressor effect was increased by concomitant ingestion of water (Jordan et al. 2004).

*Quality of evidence* Based on available data, the quality of evidence for phenylpropanolamine and pseudoephedrine to increase BP in the context of cardiovascular autonomic failure is very low.

*Safety issues* Possible side effects include supine hypertension, anxiety, tremulousness, cerebral and cardiovascular events (Freeman 2008).

*Recommendation* In consideration of quality of evidence and safety issues, recommendation for phenylpropanolamine and pseudoephedrine for the treatment of nOH is weak.

##### Octreotide

Various studies have been identified that investigate the efficacy of octreotide, a somatostatin analog, for treatment of nOH.

Although Hoeldtke and Israel 1989 observed a beneficial response to octreotide administration (0.2–26 μg/kg) in 28 patients with PAF, MSA and diabetes mellitus, inconsistency among the different study protocols applied, limits interpretation of results (Hoeldtke and Israel 1989).

Bordet et al. (1995) studied the effect of acute administration of octreotide (100 μg) in nine patients with MSA in an randomized, placebo-controlled, double-blind study. Octreotide increased duration of head-up tilt test and time until minimal BP values was reached. Notably, octreotide also raised supine BP values (Bordet et al. 1995).

The effect of octreotide on exercise-induced hypotension in the supine position was evaluated by Smith et al. (1995), demonstrating no improvement of it. Nonetheless, octreotide improved orthostatic BP fall pre- and post-exercise (Smith et al. 1995).

Data for administration of octreotide on a regular basis are limited. In an open-label study, Bordet et al. 1994 evaluated the effect of a 6-month octreotide administration in 5 patients with MSA, reporting beneficial effects (Bordet et al. 1994).

*Quality of evidence* The quality of evidence for octreotide to reduce orthostatic BP fall is low.

*Safety issues* Side effects include gastrointestinal symptoms, in particular nausea and cramps, facial flushing and hyperglycemia. For this reason, use of octreotide is not recommended in patients with diabetes mellitus, especially in case of concomitant gastroparesis diabeticorum. Supine hypertension has been also reported.

*Recommendation* Recommendation for octreotide to treat nOH is weak.

##### Other agents

For a wide range of other compounds, scattered evidence is available. Indomethacin, an inhibitor of prostaglandin synthesis, was studied with different study protocols by Jordan et al. (1998) in 35 patients with nOH of diverse etiologies. Indomethacin (50 mg) elicited a profound pressor effect. In the same study, no significant difference compared to placebo could be demonstrated for ibuprofen, caffeine and methylphenidate (Jordan et al. 1998).

The effect of the vasopressin analog, desmopressin (2–4 μg, intramuscular administration), on nocturnal polyuria was measured in a small cohort of five patients with autonomic failure. Desmopressin reduced nocturnal polyuria and prevented early morning orthostatic BP fall, but also increased supine BP (Mathias et al. 1986). Similarly, Sakakibara et al. (2003) showed that intranasal desmopressin reduced nocturia in three patients with MSA; in this study no hypertension was observed (Sakakibara et al. 2003).

*Quality of evidence* The quality of evidence for the following compounds to reduce nOH is very low: indomethacin, ibuprofen, caffeine, methylphenidate and desmopressin.

*Safety issues* Based on limited data, safety issues remain unclear.

*Recommendation* Recommendation for indomethacin, ibuprofen, caffeine, methylphenidate and desmopressin for treatment of nOH is weak.

#### Postprandial hypotension

##### α-Glucosidase inhibitor

Acarbose decreases release of vasodilatory gastrointestinal hormones through inhibition of α-glucosidase in the small intestine (Shibao et al. 2007b).

The effect of acarbose in treatment of postprandial hypotension has been evaluated in two randomized placebo-controlled trials in patients with PAF or PD (Shibao et al. 2007b) and patients with type 2 diabetes mellitus (Madden et al. 2015).

Acarbose (50–100 mg) was shown to significantly improve postprandial systolic BP fall compared to placebo. Limitation of these studies included a small sample size (*n* = 9–15) and short-term use (single administration) of acarbose.

Favorable effects were also demonstrated in an open-label study in patients with MSA (*n* = 14) (Fukushima et al. 2013) and case reports (type 1 diabetes mellitus) (Maule et al. 2004) (type 2 diabetes mellitus) (Yamamoto et al. 2006; Sasaki et al. 2001).

Voglibose, another α-glucosidase inhibitor, inhibited postprandial hypotension in a small-sized study (*n* = 11) with pre-post design in MSA, PD, diabetes mellitus and elderly patients (Maruta et al. 2006).

*Quality of evidence* The quality of evidence for acarbose to reduce postprandial BP fall is moderate, for voglibose is very low.

*Safety issues* Most frequent adverse events include gastrointestinal symptoms (e.g. flatulence, diarrhea, stomachache), which have been reported in a meta-analysis, where α-glucosidase inhibitors were administered in patients with type 2 diabetes mellitus for glycemic control (Van de Laar et al. 2005). In abovementioned studies, hypoglycemia was not reported either in patients with or without diabetes mellitus

*Recommendation* In consideration of quality of evidence, efficacy and safety profile, acarbose is strongly recommended to improve postprandial hypotension in patients with autonomic failure. Based on quality of evidence, the recommendation is weak for voglibose.

##### Octreotide

Octreotide, a somatostatin analog, combats postprandial hypotension by reducing postprandial splanchnic hyperemia induced by gastrointestinal vasodilatory peptides (Raimbach et al. 1989).

Three randomized controlled trials (Hoeldtke et al. 1986a, 1989a, 1998) have been conducted in patients with autonomic failure (mainly due to PAF, MSA or diabetes mellitus) investigating the effect of octreotide in a dosage ranging from 0.1 to 0.6 μ/kg/day on postprandial hypotension. Although these studies were limited by a small sample size (*n* = 8–16), a favorable effect of the somatostatin analogue was shown in the short-term.

Hoeldtke et al. (1986a, 1989a, b, 1998) demonstrated in all three studies that somatostatin prevented postprandial BP fall. These findings were replicated in further interventional studies with open-label design (Raimbach et al. 1989; Alam et al. 1995; Armstrong and Mathias 1991) and one case report (Hoeldtke et al. 1986b).

*Quality of evidence* Based on available data, the quality of evidence for octreotide to reduce postprandial BP fall is moderate.

*Safety issues* Side effects include gastrointestinal symptoms, in particular nausea and cramps, facial flushing and hyperglycemia. For this reason, use of octreotide is not recommended in patients with diabetes mellitus, especially in case of concomitant gastroparesis diabeticorum. Supine hypertension has been also reported.

*Recommendation* In consideration of moderate quality of evidence, efficacy and safety profile, recommendation is strong for octreotide to treat postprandial hypotension in patients with autonomic failure, with the exception of diabetes mellitus.

##### Caffeine

Caffeine inhibits adenosine-induced splanchnic vasodilatation and should therefore diminish BP fall after food ingestion (Onrot et al. 1985).

The effect of caffeine on postprandial hypotension has been evaluated only in one small-sized randomized controlled trial (*n* = 5) in patients with autonomic failure (diabetes mellitus, PAF, autonomic neuropathy due to alcoholism). Patients were treated with either caffeine 250 mg, subcutaneous dihydroergotamine (10μg/kg), a combination of both or placebo: whereas combination of treatments prevented patients from postprandial hypotension, monotherapy improved postprandial BP fall only partially (Hoeldtke et al. 1989b).

Onrot et al. (1985) further studied the effect of caffeine in a dosage of 250 mg in a small cohort (*n* = 6) of patients with autonomic failure. In this study, which did not include a placebo arm, caffeine showed a favorable single-dose effect on postprandial hypotension, which was maintained after 7-day administration (Onrot et al. 1985).

*Quality of evidence* Based on available literature, quality of evidence for caffeine to treat postprandial hypotension is very low.

*Safety issues* No side effects reported.

*Recommendation* Based on very low quality of evidence, the recommendation for treating post-prandial hypotension with caffeine is weak.

#### Remarks on classification of evidence

Tables 1, 2, 3 summarize reviewed studies for non-pharmacological and pharmacological management of nOH and post-prandial hypotension, while Table 4 provides an overview of quality of evidence, recommendation and safety issues.

**Table 1:** Non-pharmacological interventions for treatment of nOH and post-prandial hypotension—available studies

Compound	Dosage	Schedule	Study design	Sample size	Patients	Duration	Outcome measures	Results	Safety issues	Author
**Water**										
Water Liquid meal	300 ml 300 ml (550 kcal)	1 × 1 1 × 1	Open Supine BP measurement before and continued for a time of 45 min after liquid meal/water ingestion, followed by HUT	17	MSA PAF DBH	1 day	Supine BP BP during HUT	Supine and HUT BP values were lower in MSA and PAF patients after liquid meal ingestion Water had no effect on BP (either supine or during HUT)	No adverse effects reported	Mathias et al. (1991)
Water	480 ml	1 × 1	Open Seated BP measurement before and continued for a time of 90 min after water drinking	19	PAF MSA	1 day	SBP DBP HR Plasma volume Plasma vasopressin PRA	Significant rise in SBP (*p* < 0.001) 35 min after water ingestion	No adverse effects reported	Jordan et al. (1999)
Water	500 ml	1 × 1	Open Supine BP measurement before and 60 min after water drinking, followed by standing test	13	PD	1 day	Supine BP SBP/DBP and HR changes during standing test	No significant effect on supine BP No change in orthostatic BP behavior	No adverse effects reported	Senard et al. (1999)
Water	480 ml	1 × 1—quick ingestion	Open Seated BP measurement before and continued for a time of 90 min after water drinking	47	MSA PAF	1 day	Change in seated SBP/DBP and HR	Significant increase in BP (*p* < 0.0001) and HR (*p* < 0.001) Maximum was reached after 30 to 35 min after water drinking	No adverse effects reported	Jordan et al. (2000)
Distilled water	500 ml	1 × 1—in 3–4 min	Open Seated BP measurement before and for 60 min after water ingestion	14	PAF	1 day	SBP DBP HR TPR SV EF CO	Significant SBP and DBP increase after water ingestion (*p* < 0.001) BP plateau reached 10–35 min after water ingestion, with a peak at 14 min Effect decay after 50 min	No adverse effects reported	Cariga and Mathias (2001)
Water			Open	18	MSA PAF	1 day	Protocol 1 change in seated and upright SBP/DBP (1-min after standing) tolerated standing time	Significant increase in seated (*p* < 0.001) and standing (*p* < 0.01) BP after water ingestion	No adverse effects reported	Shannon et al. (2002)
Protocol 1										
Water	480 ml	1 × 1—in < 5 min	Seated BP measurement before and for 35 min after water ingestion, followed by standing test							
Protocol 2										
Water	480 ml	1 × 1—immediately before test meal	Seated BP measurement before and for 90 min postprandial				Protocol 2 change in postprandial SBP	Significant increase in postprandial BP (*p* < 0.001) after water drinking		
Distilled water	480 ml	1 × 1—in < 5 min	Open Seated and standing BP measurement before, 15 and 35 min after water ingestion	14	MSA PAF	1 day	Change in seated and standing SBP/DBP	Significant rise in seated and standing BP (*p* < 0.05) 15 and 35 min after water ingestion	No adverse effects reported	Young and Mathias (2004)
Water followed by standardized breakfast at 45 min later	350 ml	1 × 1—in < 5 min	Open label, controlled, no randomization Seated BP measurement before and for 30 min after water ingestion followed by standing test After standardized breakfast seated BP measurement was continued for 90 min	5	MSA	7 days Day 0 without water drinking Day 1–7 with water drinking	Change in SBP, DBP (seated, standing, postprandial; compared for day 0, 1,7) adverse effects	Seated and standing BP rise after water ingestion (*p* < 0.05) Reduced postprandial BP fall in case of water ingestion (*p* < 0.05)	No severe adverse effects reported	Deguchi et al. (2007)
Distilled water	480 ml	1 × 1—immediately after pre-exercise standing	Open, controlled, no randomization Protocol consisted of 5 min standing test, 10 min supine rest, 9 min supine exercise,10 min supine rest and 5 min standing test	8	PAF	1 day each protocol (with and without water)	Absolute time of standing pre- and post-exercise maximally achieved exercise capacity total exercise time hypotension related symptoms during standing comparison of symptoms between 1st and 2nd standing	Significant supine BP (*p* < 0.05) rise after water ingestion Similar BP fall during supine exercise with and without water drinking Post-exercise BP was significantly (p < 0.05) higher with water drinking	No adverse effects reported	Humm et al. (2008)
**Sodium intake**										
Saline	500 ml 0.9% NaCl	1 × 1	Single-blinded, crossover fashion	10	MSA	1 day	Change in SBP DBP	No change in BP after saline administration	No adverse events reported	Lipp et al. (2005)
Distilled water	500 ml	1 × 1 Through nasogastric tube over 5 min	Randomization unclear				HR	Water led to increase in BP within 10 min that reached maximum after 20 min Significant (*p* = 0.02) difference in BP (20 min post-administration) after water administration with respect to saline		
Saline	2 g in 473 ml H_2_O	1 × 1	Open, randomized crossover fashion	9	OH	1 day each intervention	Primary: AUC for SBP change (30 min post-administration; seated) Secondary: AUC for SBP change (60 min post-administration; seated) change in seated SBP, DBP, HR (at 30 and 60 min) SBP, DBP, HR compared with each intervention NE and epinephrine levels	AUC for SBP was significant greater for water alone compared with NACL (30 min *p* < 0.002; 60 min *p* = 0.048) Significant increase in SBP after ingestion of saline at 30 min (*p* < 0.05) Significant increase in SBP after water intake (at 30 min and 60 min) (*p* < 0.05) Increase in SBP was significant greater at 30 min (*p* = 0.006) with water alone compared with NACL	No adverse effects reported	Raj et al. (2006)
Distilled water	473 ml	1 × 1								
Clear soup	450 ml (1.7 g salt)	1 × 1	Open, randomized, crossover fashion	7 7	MSA POTS	1 day each intervention	Change in supine, HUT 3 min and 5 min hemodynamic parameters: SBP DBP HR SV CO TPR	Premature termination of HUT in 2 (water) and 6 (soup) MSA patients Increase in SBP/DBP (supine and upright) after water drinking and decrease after ingestion of clear soup Significant difference between water and soup intake in consideration of BP change (p < 0.05) No significant difference in any other hemodynamic parameters	No adverse effects reported	Z’Graggen et al. (2010)
Water	450 ml	1 × 1								
**Meal size**										
Large meal	2.5 MJ/day	3 × 1	Open, randomized, crossover fashion	7	MSA PAF	1 day each intervention	BP (30 min postprandial) supine seated upright	Larger meals resulted in a significant lower BP in all positions compared with smaller meals (*p* < 0.05)	No adverse effects reported	Puvi-Rajasingham and Mathias (1996)
Small meal	2.5 MJ/day	6 × 1				At least 1 day apart	OH-related symptoms	The mean fall in orthostatic BP was unaffected Large meals resulted in significant lower BP levels (*p* < 0.05) between meals Large meals were associated with more frequent symptoms of OH		
**Head-up tilt position**										
HUT (12°) alone			Open	6	Hypoadrenergic OH	1 w each treatment arm W1: control phase W2: HUT alone W3: HUT in combination with fludrocortisone Follow-up: 14 months	Orthostatic tolerance maximum standing time orthostatic BP fluid balance	Combination of treatment significantly decreased orthostatic dizziness (*p* < 0.001) and led to increase in standing time Significantly reduced SBP drop (1-min upright) with HUT alone (*p* < 0.01) and in combination with fludrocortisone (p < 0.05) Standing BP after 1 min significantly increased with combination therapy (*p* < 0.05) No influence on supine BP	Head-up tilt none Fludrocortisone hypokalemia ankle edema	Ten Harkel et al. (1992)
HUT (12°) + fludrocortisone	0.1–0.2 mg	At bedtime								
HUT (12°) in combination with fludrocortisone	0.1–0.2 mg	At bedtime	Open	8	PAF others	3 w	Protocol 1 (*n* = 6) orthostatic BP Protocol 2 (*n* = 4) BP HR packed cell volume PRA aldosterone level antidiuretic hormone level ANP	Significant (*p* < 0.05) increase in SBP (1 min after standing) compared to pre-treatment Minimal improvement of orthostatic tolerance in standing position No influence on supine BP by treatment	Edema	Van Lieshout et al. (1999)
**Physical counter maneuvers**										
1. Leg crossing 2. Squatting			Open	7	PAF others	1 day	Change in orthostatic BP SBP DBP MBP	Leg crossing and squatting improved OH by increasing BP	No adverse effects reported	Van Lieshout et al. (1992)
1. Leg crossing 2. Tiptoeing			Open, randomized Baseline without countermaneuver	7	PAF others	1 day	Change in orthostatic BP (SBP/DBP/MBP) change in HR change in SV change in CO change in SVR	Significant increase in BP (*p* < 0.05) for leg crossing Tiptoeing had no effect on orthostatic BP in patients	No adverse effects reported	Ten Harkel et al. (1994)
Physical countermaneuvers 1. Squatting 2. Genuflection-contraction 3. Leg crossing 4. Knee flexion 5. Toe raise 6. Neck flexion 7. Abdominal contraction 8. Thigh contraction 9. Combination Biofeedback training			Open, randomized Baseline without countermaneuver	9	PAF MSA AN	1 day 3–4 months (long term assessment)	Global symptomatic improvement score orthostatic BP continued improvement	Most of the countermaneuvers led to significant increase (*p* < 0.05) in SBP (knee and neck flexion, leg crossing, combination, squatting, toe raise) Biofeedback training augmented efficacy of genuflection-contraction, leg crossing, thigh contraction Regular application led to improvement of symptoms	No adverse effects reported	Bouvette et al. (1996)
Protocol 1 (*n* = 7) Abdominal compression (G-suit; 40 mmHg)			Open	23	PAF MSA DM unknown etiology	1 day	Protocol 1 orthostatic BP change (SBP/DBP/HR) ΔSV ΔCO, ΔTPR Vein diameters Protocol 2 standing BP Protocol 3 standing BP	Abdominal compression (40 mmHg led) to significant increase (*p* < 0.05) of upright BP All countermeasures improved standing BP compared to baseline (*p* < 0.05) 40 mmHg abdominal compression produced greater pressure response than 20 mmHg (*p* < 0.05) Abdominal compression with leg crossing was more effective than standing with abdominal compression alone (*p* < 0.05) Significant increase in standing BP with elastic binder (*p* < 0.05)	No adverse effects reported	Smit et al. (2004)
Protocol 2 (*n* = 12) Six standing session 1. Abdominal compression 20 mmHg 2. Abdominal compression 40 mmHg 3. Leg crossing 4. Combination abdominal compression 20 mm and leg crossing 5. Combination abdominal compression 40 mm and leg crossing			Open, randomized Baseline without compression							
Protocol 3 (*n* = 9) Elastic abdominal compression (15–20 mmHg)			Open, randomized							
Bending forward Squatting Leg crossing Abdominal compression (20 mmHg)			Open, randomized Baseline without compression	17	FD	1 day	Orthostatic BP (SBP/DBP/MBP/HR) SV CO TPR Calf volume	Significant increase in mean BP was shown for squatting (*p* = 0.002) bending forward (*p* = 0.005) abdominal compression (*p* = 0.04) not for leg crossing	No adverse effects reported	Tutaj et al. (2006)
**Abdominal binder/compression stockings**										
G-suit with five different compartments (40 mmHg): 1. Bilateral calves 2. Bilateral thighs 3. Combination 1 + 2 4. Low abdomen 5. Combination of all			Open, randomized Baseline without compression	14	MSA PAF DM	1 day	Symptomatic improvement of OH (verbal scale, visual analog scale) orthostatic BP change (SBP/DBP/MBP) preload (EDI/stroke index) afterload (PRI)	Compression of all compartments and of abdomen alone led had a significant effect on orthostatic BP compared to baseline (*p* < 0.01) Symptoms of OH improved according to the following: 5 > 4>3 > 3=1 > 1	No adverse effects reported	Denq et al. (1997)
Protocol 1 (n = 7) Abdominal compression (G-suit; 40 mmHg)			Open	23	PAF MSA DM unknown etiology	1 day	Protocol 1 orthostatic BP change (SBP/DBP/HR) ΔSV ΔCO, ΔTPR Vein diameters Protocol 2 standing BP Protocol 3 standing BP	Abdominal compression (40 mmHg led) to significant increase (*p* < 0.05) of upright BP All countermeasures improved standing BP compared to baseline (*p* < 0.05) 40 mmHg abdominal compression produced greater pressure response than 20 mmHg (*p* < 0.05) Abdominal compression with leg crossing was more effective than standing with abdominal compression alone (*p* < 0.05) Significant increase in standing BP with elastic binder (*p* < 0.05)	No adverse effects reported	Smit et al. (2004)
Protocol 2 (*n* = 12) Six standing session 1. Abdominal compression 20 mmHg 2. Abdominal compression 40 mmHg 3. leg crossing 4. Combination abdominal compression 20 mm and leg crossing 5. Combination abdominal compression 40 mm and leg crossing			Open, randomized Baseline without compression							
Protocol 3 (*n* = 9) Elastic abdominal compression (15–20 mmHg)			Open, randomized							
1. Bending forward 2. Squatting 3. Leg crossing 4. Abdominal compression (20 mmHg)			Open, randomized Baseline without compression	17	FD	1 day	Orthostatic BP (SBP/DBP/MBP/HR) SV CO TPR Calf volume	Significant increase in mean BP was shown for squatting (*p* = 0.002) bending forward (*p* = 0.005) abdominal compression (*p* = 0.04), but not for leg crossing	No adverse effects reported	Tutaj et al. (2006)
Abdominal compression (conventional and adjustable binders 1. Abdominal binding prior to rising (10 mmHg) 2. Upright maximal compression 3. Upright comfortable compression			Open, randomized, crossover trial Baseline without compression	13	PAF MSA PD others	1 day	Primary postural changes in SBP Secondary subject assessments of preference ease of use	Mild abdominal (conventional and adjustable) compression significantly (*p* = 0.03) decreased SBP drop Increasing compression to maximal tolerance did not provide additional benefit on OH Decompression tended to worsen OH	No adverse effects reported	Figueroa et al. (2015)
Abdominal binder (20 mmHg)			Single-center, single-blind, randomized placebo-controlled, crossover study Open-label follow-up	15	PD	1 day each intervention 1 day apart Follow-up: 4 w	Primary mean BP changes upon head-up tilt (after 3 min) Secondary SBP, DBP change upon head-up tilt (after 3 min) mean BP change upon standing test (after 3 min) mean supine BP changes of the OHQ score (after 4 weeks)	Abdominal compression significantly reduced BP fall upon tilt table (*p* = 0.006) compared to placebo No significant effect on supine blood pressure compared to placebo was measured (*p* = 0.3) During 4-week follow-up, symptoms significantly improved (*p* = 0.003).	No adverse effects reported	Fanciulli et al. (2016a)
**Various non-pharmacological options**										
Phase I 1. Increased salt/fluid intake 2. Elevated head of bed 3. Compression stockings (30 mm Hg) 4. Small meals (6/day) 5. Avoidance of triggers 6. Other			Open	17	PD	Phase I 3 w	Compliance postural BP COMPASS-OD CGI	The overall compliance was 78% Postural BP did not significantly change No significant change in COMPASS-OD and CGI was demonstrated	None	Schoffer et al. (2007)
Phase II			Phase II Two-center, double-blind, randomized crossover trial No placebo			Phase II 3 w each treatment arm 1 w of washout phase	Primary COMPASS-OD CGI postural BP	Fludrocortisone (*p* = 0.02) and domperidone (*p* = 0.04) improved COMPASS-OD scores Both treatment arms improved CGI (no statistical comparisons available) A trend towards reduced orthostatic BP fall was shown	Fludrocortisone: nausea chest discomfort headache lightheadedness dizziness Domperidone nausea chest pain abdominal pain palpitations headache	
Fludrocortisone Domperidone	0.1 mg 10 mg	1 × 1 TID								

*AN* autonomic neuropathy, *ANP* atrial natriuretic peptide, *AUC* area under the curve, *CGI* clinical global impression of change, *CO* cardiac output, *COMPASS-OD* Composite Autonomic Symptom Scale, *DM* diabetes mellitus, *DBH* dopamine beta hydroxylase deficiency, *DBP* diastolic blood pressure, *EDI* end-diastolic index, *EF* ejection fraction, *FD* familial dysautonomia, *HR* heat rate, *HUT* head-up tilt, *MBP* mean blood pressure, *MSA* multiple system atrophy, *min* minutes, *NACl* sodium chloride, *NE* norepinephrine, *OH* orthostatic hypotension, *OHQ* orthostatic hypotension questionnaire, *PAF* pure autonomic failure, *PoTS* postural tachycardia syndrome, *PD* Parkinson’s disease, *PRA* plasma levels of renin activity, *PRI* peripheral resistance index, *SBP* systolic blood pressure, *SV* stroke volume, *SVR* systemic vascular resistance, *TID* three times a day, *TPR* total peripheral resistance, *w* week

**Table 2:** Pharmacological interventions for treatment of nOH—available studies

Compound	Dosage	Schedule	Study design	Sample size	Patients	Duration	Outcome measures	Results	Safety issues	Author
**Midodrine**										
Midodrine alone	25–40 mg/day	4 × 1	Open	7	MSA PAF	4–8 days of titration phase of midodrine Maintenance period for 1 week w each treatment arm 2 days wash-out phase	BP and HR (2-h interval) supine BP upright BP (2 min after standing) mean arterial BP body weight quantification of respiratory sinus arrhythmia E/I ratio PRA, sodium, potassium, creatinine measured supine and upright	Midodrine significantly (*p* < 0.05) increased upright BP in group I (responders) Treatment with fludrocortisone alone or in combination with midodrine elevated upright BP in group I In group II (non-responder) midodrine significantly (*p* < 0.05) decreased upright BP Significant decrease in upright BP in group II with fludrocortisone	Midodrine Led to significant increase in supine BP in group II, not in group I Scalp pruritus	Kaufmann et al. (1988)
Midodrine in combination with	25–40 mg/day	4 × 1	Single-center, randomized, placebo-controlled, double-blind, crossover fashion							
Fludrocortisone or fludrocortisone alone	0.1 mg	1 × 1								
Midodrine	2.5 mg 5 mg 10 mg	TID	Multi-center, randomized, double-blind, placebo-controlled, parallel group	97	PAF MSA DM PD others	1-week single-blind placebo run-in phase 4-week randomized, placebo-controlled phase	Primary standing SBP (1 min after standing; 1-h post-dose) symptoms of OH Secondary standing DBP supine SBP and DBP supine and standing HR	Significant increase in standing SBP (*p* < 0.001) and improvement of OH related symptoms OH (*p* < 0.05) with respect to placebo	SH Urinary urgency scalp Pruritus/tingling Headache	Jankovic et al. (1993)
Midodrine	2.5–10 mg	TID	Single-center, placebo-controlled randomized, double-blind, blocked, crossover fashion	8	PAF MSA	2-day placebo run-in period (single-blind) Double-blind titration and maintenance period of 3–5 days each 4 days placebo wash out	Supine mean SBP, DBP, HR standing mean SBP, DBP, HR (after 1 min) ability to stand incidence of SH	Midodrine significantly improved standing SBP and DBP compared to ephedrine (p < 0.05) and placebo (*p* < 0.001) Ephedrine did not significantly increase standing BP (*p* > 0.05) compared to placebo Ephedrine (*p* < 0.01) and midodrine (*p* < 0.001) significantly increased supine BP compared to placebo without any difference between the two treatment arms Midodrine (*p* < 0.01), but not ephedrine led to an increased ability to stand	Midodrine SH Scalp pruritus Urinary retention Ephedrine SH Dizziness/lightheadedness Photosensitivity Disequilibrium	Fouad-Tarazi et al. (1995)
Ephedrine	6–24 mg	TID								
Midodrine	10 mg	TID	Multi-center, randomized, double-blind, placebo-controlled, parallel group	171	PAF MSA PD DM others	w placebo run-in phase (single-blind) w double-blind period 2 w single-blind placebo washout phase	Primary standing SBP (after 1 min, 1 h post-dose) symptoms of light headedness Secondary global assessment	Midodrine significantly improved standing SBP(p < 0.001), reported symptoms of OH (p = 0.02) and global assessment (p < 0.05) with respect to placebo	SH Urinary retention Piloerection Scalp pruritus Paresthesia Chills	Low et al. (1997)
Midodrine	2.5 mg 10 mg 20 mg	1 × 1	Two-center, placebo-controlled, randomized, double-blind, crossover fashion	25	PAF MSA DM PD	1 day each treatment arm Conducted on successive days (day 2–5)	Primary 1-h postdose standing SBP (after 1 min) Secondary symptom response safety duration of midodrine action desglymidodrine levels	Significant increase in standing SBP for 10 and 20 mg (*p* < 0.05) Midodrine dose and mean SBP were linearly related) Significant improvement of symptoms for 10 and 20 mg (p < 0.05)	SH Piloerection Pruritus Paresthesia	Wright et al. (1998)
Midodrine	5–10 mg	1 × 1	Single-center, randomized, single-blind, placebo-controlled, crossover design	69	PAF PD MSA	1 day each treatment arm Unclear if conducted on consecutive days	Primary post-dose upright SBP at 1 min Secondary post-dose seated SBP and DBP upright DBP and HR OHQ and Q1 symptom scores	Both midodrine and atomoxetine significantly (*p* ≤ 0.001) increased upright SBP and DBP compared with placebo Compared to midodrine, atomoxetine led to a higher increase in upright SBP (*p* = 0.03) and DBP (*p* = 0.05) Both atomoxetine and midodrine significantly increased (p < 0.001) seated SBP and DBP with respect to placebo Only atomoxetine (*p* = 0.02) improved symptoms of OH based on total OHQ score compared to placebo	Supine BP not measured	Ramirez et al. (2014)
Atomoxetine	18 mg	1 × 1								
**Droxidopa**										
Droxidopa	1000 mg	1 × 1	Single-center, randomized, double-blind, placebo-controlled, crossover trial	10	MSA PAF	1 day each treatment arm On consecutive days	Primary change in supine and upright SBP change in supine and upright DBP Secondary change in plasma levels of NE and DL-DOPS forearm vascular resistance quality of life	Increase in SBP (supine *p* < 0.001; upright *p* < 0.05) and DBP (both *p* < 0.01) Trend towards improvement of OH-related symptoms	SH	Freeman et al. (1999)
Droxidopa	200–2000 mg	1 × 1	Two-center, randomized, double-blind, placebo-controlled, crossover trial	19	MSA PAF	Initial dose-ranging phase 1 day each treatment arm 1 day wash-out	Primary supine BP standing BP (after 1 and 3 min) supine HR standing HR (after 1 and 3 min) orthostatic tolerance Secondary plasma levels of NE and L-DOPS	Significant increased BP (supine and standing; *p* < 0.001) with respect to placebo Significant improvement of orthostatic tolerance (*p* < 0.001)	SH Hyponatremia Transient anginal pain	Kaufmann et al. (2003)
Droxidopa	100–600 mg	TID	Multi-center, randomized, double-blind, placebo-controlled, parallel group, phase 3	162	MSA PAF PD NDAN	2 w dose optimization phase (open) 1 w wash-out 1 w treatment period	Primary change in overall OHQ composite score (randomization to study end) Secondary change in OHSA composite score change in OHDAS composite score individual QHQ items Tertiary change in standing SBP (3 min after standing; randomization to study end)	Significant improvement in OHQ composite score (*p* = 0.003) with respect to placebo Droxidopa significantly improved OHSA (*p* < 0.05) and OHDAS score (*p* < 0.01) Significant increase in mean standing SBP (*p* < 0.001) compared with placebo	SH Headache Dizziness Fatigue Syncope Gastrointestinal Complaints Urinary tract symptoms	Kaufmann et al. (2014)
Droxidopa	100–600 mg	TID	Multi-center, randomized, double-blind, placebo-controlled, parallel group, phase 3	101	MSA PAF PD NDAN Dopamine-β-hydroxylase deficienc others	2 w dose optimization phase (open) 1 w open-label treatment 2 w treatment period (randomized, placebo-controlled)	Primary change in OHSA item 1 Secondary change in other 5 OHSA items change in the 4 OHDAS ratings CGI	Droxidopa showing no difference in OHSA item 1 compared to placebo failed to meet primary endpoint (*p* = 0.509) OHSA and OHDAS ratings favored droxidopa with statistical significance for OHDAS item 1 and 2	SH Headache Dizziness Fatigue Falls Gastrointestinal Symptoms Urinary tract Symptoms	Biaggioni et al. (2015)
Droxidopa nOH306A	100–600 mg	TID	Multi-center, randomized, double-blind, placebo-controlled, parallel group, phase 3 trial	51	PD	≤ 2 w of dose optimization phase 8 w of maintenance period	Primary mean change in OHQ composite score (from baseline to study end) Secondary OHQ item 1 patient-reported falls OHSA composite score OHDAS composite score QHQ individual items supine SBP standing SBP	Pre-planned interim efficacy analysis showed no difference in change of OHQ composite score with respect to placebo Difference in standing systolic BP was significant at week 1 (*p* < 0.05) Primary outcome was subsequent changed	SH Nausea Falls Urinary tract Symptom Dizziness	Hauser et al. (2014)
Droxidopa nOH306B	100–600 mg	TID	Multi-center, randomized, double-blind, placebo-controlled, parallel group, phase 3 trial	171	PD	≤ 2 w of dose optimization phase 8 w of maintenance period	Primary change in OHSA score item 1 (at 1 w) Secondary mean change on OHSA item 1 (baseline to w 2, 4, 8) mean change in lowest standing SBP between 0 and + 3 min of standing (baseline to w 1) patient reported falls (baseline to w 8) mean change in OHQ composite score (baseline to w 8)	Droxidopa significantly improved (*p* = 0.018) primary endpoint measured by OHSA score item 1 at 1 week Change in OHSA item 1 from baseline to weeks 2, 4, 8 did not significantly differ from placebo the change in lowest standing SBP from baseline to week 1 was significant with respect to placebo	SH Headache Dizziness Fatigue Nausea	Hauser et al. (2015)
**Fludrocortisone**										
Midodrine alone	25–40 mg/d	4 × 1	Open Single-center, randomized, placebo-controlled, double-blind, crossover fashion	7	MSA PAF	4–8 days of titration phase of midodrine Maintenance period for 1 week 1 w each treatment arm 2 d wash-out phase	BP and HR (2-h interval) supine BP upright BP (2 min after standing) mean arterial BP body weight quantification of respiratory sinus arrhythmia E/I ratio PRA, sodium, potassium, creatinine measured supine and upright	Treatment with fludrocortisone alone or in combination with midodrine elevated upright BP in group I (responders) Significant decrease in upright BP in group II with fludrocortisone (non responder) Midodrine significantly (p < 0.05) increased upright BP in group I In group II midodrine significantly (p < 0.05) decreased upright BP	Fludrocortisone Not mentioned Midodrine Led to significant Increase in supine BP in group II, not in group I Scalp pruritus	Kaufmann et al. (1988)
Fludrocortisone alone or in combination with	0.1 mg	1 × 1								
Midodrine	25–40 mg/day	4 × 1								
Phase II			Phase II Two-center, double-blind, randomized crossover trial No placebo	17	PD	Phase II 3 w each treatment arm 1 w of washout phase	Primary COMPASS-OD CGI postural BP	Fludrocortisone (*p* = 0.02) and domperidone (*p* = 0.04) improved COMPASS-OD scores Both treatment arms improved CGI (no statistical comparisons available) A trend towards reduced orthostatic BP fall was shown	Fludrocortisone: Nausea Chest discomfort Headache Lightheadedness Dizziness Domperidone Nausea Chest pain Abdominal pain Palpitations Headache	Schoffer et al. (2007)
Fludrocortisone	0.1 mg	1 × 1								
Domperidone	10 mg	TID								
Phase I Increased salt/fluid intake Elevated head of bed Compression stockings (30 mm Hg) Small meals (6 per day) Avoidance of triggers Other			Open			3 w	Compliance Postural BP COMPASS-OD CGI	The overall compliance was 78% Postural BP did not significantly change No significant change in COMPASS-OD and CGI was demonstrated	None	
**Pyridostigmine**										
Pyridostigmine alone/or in	60 mg	1 × 1	Single-center, randomized double-blind, placebo-controlled, cross-over fashion	58	MSA PAF DAN AAN unspecified nOH	1 day each treatment arm Conducted on successive days	Primary improvement of standing DBP 1 h post-dose Secondary influence on SBP and supine BP relation of symptoms to BP change predictors of treatment response influence on plasma NE, epinephrine, dopamine levels	In consideration of primary endpoint, pyridostigmine significantly (*p* = 0.02) differed from placebo Pyridostigmine alone (*p* = 0.04) and in combination with midodrine 5 mg (p = 0.002) led to significant reduction of BP drop compared with placebo	Supine BP did not differ significantly between groups	Singer et al. (2006b)
Combination with midodrine	2.5 mg or 5 mg	1 × 1								
Pyridostigmine	60 mg	1 × 1	Single-center, single-blind, randomized, placebo-controlled, crossover fashion	31	MSA PAF PD	1 day each treatment arm Unclear if conducted on consecutive days	Primary change in standing DBP 1 h post-dose Secondary orthostatic Symptom score change in seated SBP/DBP 1 h post-dose	Yohimbine led to significant increase in standing DBP (*p* < 0.001) 1 h post-dose Pyridostigmine did not significantly increase standing DBP (*p* = 0.823) Only yohimbine significantly (*p* = 0.006) improved OH symptoms compared with placebo Yohimbine, but not pyridostigmine, significantly (*p* < 0.05) increased seated SBP and DBP In patients with central autonomic failure increase in seated BP was significantly higher than in patients with peripheral autonomic failure with yohimbine Combination of treatment did not show synergistic pressor effect	Supine BP not measured	Shibao et al. (2010)
Yohimbine	5.4 mg	1 × 1								
Combination (*n* = 16)										
**Yohimbine**										
Yohimbine	2 mg	TID	Single-center, double-blind, placebo-controlled, randomized, crossover fashion	17	PD	Weeks for each treatment sequence Wash-out phase unclear	mean SBP (after 4 w) mean DBP (after 4 w) mean HR (after 4 w) BP variability (after 4 w) nychtemeral rhythm (after 4 w)	No significant difference in mean SBP, DBP, HR, BP variability, nychtemeral rhythm with respect to placebo or baseline	No serious side effects reported	Senard et al. (1993)
Yohimbine	5.4 mg	1 × 1	Single-center, single-blind, randomized, placebo-controlled, crossover fashion	31	MSA PAF PD	1 day each treatment arm Unclear if conducted on consecutive days	Primary change in standing DBP 1 h post-dose Secondary orthostatic symptom score change in seated SBP/DBP 1 h post-dose	Yohimbine led to significant increase in standing DBP (*p* < 0.001) 1 h post-dose Pyridostigmine did not significantly increase standing DBP (*p* = 0.823) Only yohimbine significantly (*p* = 0.006) improved OH symptoms compared with placebo Yohimbine, but not pyridostigmine, significantly (*p* < 0.05) increased seated SBP and DBP In patients with central autonomic failure increase in seated BP was significantly higher than in patients with peripheral autonomic failure with yohimbine Combination of treatment did not show synergistic pressor effect	Supine BP not measured	Shibao et al. (2010)
Pyridostigmine	60 mg	1 × 1								
Combination (*n* = 16)										
Yohimbine	5.4 mg	1 × 1	Single-center, single-blind, randomized, placebo-controlled, crossover study Combination therapy was not randomized	17	PAF PD	1 day each treatment arm Unclear if conducted on consecutive days	Primary seated SBP 1-h post-drug Secondary orthostatic tolerance orthostatic symptom score	Both treatment arms neither significantly changed primary endpoint (*p* > 0.05) nor orthostatic tolerance (*p* > 0.05) compared to placebo Only the combination showed significant increase in seated SBP (*p* < 0.001) and improvement of symptoms (*p* < 0.05)	Supine BP not measured	Okamoto et al. (2012)
Atomoxetine	18 mg	1 × 1								
Combination										
**Atomoxetine**										
Atomoxetine	18 mg	1 × 1	Single-center, single-blind, randomized, placebo-controlled crossover fashion	21	MSA PD PAF	1 day each treatment arm Unclear if conducted on consecutive days	Primary mean SBP (taken for 1 h after drug administration) Secondary change from baseline in seated SBP and standing SBP at 60 min absolute values in seated SBP and standing SBP at 60 min Tertiary difference NE and the plasma dihydoxyphenylglycol to NE ratio (baseline and post-drug)	Significant increase in seated and upright SBP (*p* < 0.05) in patients with central autonomic failure in comparison with placebo In patients with peripheral autonomic failure there was no difference in seated and standing SBP with respect to placebo (*p* > 0.05)	Supine BP not measured	Shibao et al. (2007a)
Atomoxetine	18 mg	1 × 1	Single-center, single-blind, randomized, placebo-controlled, crossover study Combination therapy was not randomized	17	PAF PD	1 day each treatment arm Unclear if conducted on consecutive days	Primary seated SBP 1-h post-drug Secondary orthostatic tolerance orthostatic symptom score	Both treatment arms neither significantly changed primary endpoint (*p* > 0.05) nor orthostatic tolerance (*p* > 0.05) compared to placebo Only the combination showed significant increase in seated SBP (*p* < 0.001) and improvement of symptoms (*p* < 0.05)	Supine BP not measured	Okamoto et al. (2012)
Yohimbine	5.4 mg	1 × 1								
Combination										
Atomoxetine	18 mg	1 × 1	Single-center, randomized, single-blind, placebo-controlled, crossover design	69	PAF PD MSA	1 day each treatment arm Unclear if conducted on consecutive days	Primary post-dose upright SBP at 1 min Secondary post-dose seated SBP and DBP upright DBP and HR OHQ and Q1 symptom scores	Both treatment arms significantly (*p* ≤ 0.001) increased upright SBP and DBP compared with placebo Compared to midodrine atomoxetine led to a higher increase in upright SBP (*p* = 0.03) and DBP (*p* = 0.05) Atomoxetine and midodrine significantly, without any difference, increased (*p* < 0.001) seated SBP and DBP with respect to placebo Only atomoxetine (*p* = 0.02) improved symptoms of OH based on total OHQ score compared to placebo	Supine BP not measured	Ramirez et al. (2014)
Midodrine	5–10 mg	1 × 1								
**Ergot alkaloids**										
DHE				9	DM PAF alcoholism				DHE supine BP Caffeine not mentioned	Hoeldtke et al. (1989b)
Protocol 1		1 × 1								
DHE sc	6.5 μg/kg 13 μg/kg		Single-center, randomized, placebo-controlled, crossover fashion Blinding unclear	8		1 day each treatment arm On consecutive days	Supine mean BP upright mean BP (after 2 min) postprandial BP	Significant increase (*p* < 0.001) of upright BP and supine BP (*p* < 0.01) Average orthostatic BP drop was similar compared with placebo DHE failed to prevent postprandial hypotension		
Protocol 2										
DHE sc	10 μg/kg	1 × 1 60 min before breakfast	Single-center, randomized, placebo-controlled, crossover fashion No blinding	5		1 day each treatment arm On consecutive days	Postprandial sitting mean BP	Caffeine or DHE prevented postprandial OH only partly Combination therapy had a significant greater effect on postprandial hypotension (*p* < 0.01) and BP than monotherapy with either caffeine or DHE		
Caffeine	250 mg	1 × 1 30 min before breakfast								
Combination										
DHE				28	PAF MSA DM SOH				Combination therapy (DHE and octreotide) finger cyanosis in MSA patient Octreotide nausea and abdominal cramps in DM pain due to injection impaired glucose tolerance loss of consciousness hyperpigmentation	Hoeldtke and Israel (1989)
Protocol 4										
Octreotide sc	1.2 μg/kg	1 × 1 10 min before tilting	Single-center, placebo-controlled, randomized, crossover fashion Blinding unclear	6	PAF MSA	1 day each treatment arm Unclear if conducted on consecutive days	Upright mean BP (tilt table)	Only combination therapy significantly (*p* < 0.05) improved OH		
DHE sc	10 μg/kg	1 × 1 70 min before tilting								
Combination										
Protocol 1										
Octreotide sc	0.2 μg/kg 0.4 μg/kg	1 × 1	Single-center, placebo-controlled, randomized, crossover fashion Blinding unclear	28		1 day each treatment arm Unclear if conducted on consecutive days	Average semirecumbent BP	With exception of patients with OH, BP significantly increased (*p* < 0.05)		
Protocol 2										
Octreotide sc	0.4 μg/kg 0.8 μg/kg 1.2 μg/kg 1.6 μg/kg	1 × 1 at the end of breakfast	Single-center, placebo-controlled, randomized, single-blind, crossover design	15	PAF MSA SOH	1 day each treatment arm Unclear if conducted on consecutive days	BP during walking walking time	In MSA and PAF octreotide significantly improved walking time *p* < 0.01 and upright BP *p* < 0.05 Treatment combination (DHE and octreotide) improved upright BP only partially		
DHE sc in comination with	12 μg/kg	1 × 1 60 min before breakfast								
Caffeine	250 mg	30 min before breakfast								
Protocol 3										
Octreotide sc	1.2 μg/kg 0.3 μg/kg 0.9 μg/kg	1 × 1 bolus priming dose continuous infusion over 70 min Administered 10 min before tilt table test	Single-center, placebo-controlled, randomized, crossover fashion Blinding unclear	17	PAF MSA DM	1 day each treatment arm Unclear if conducted on consecutive days	Upright mean BP (tilt table)	Continuous and bolus sc octreotide improved tilt table tolerance Octreotide infusion significantly (*p* < 0.05) increased BP		
Protocol 5										
DHE sc and propanolol	12–20 μg/kg 30 mg	1 × 1 60–90 min before octreotide	Open	3	SOH		Walking BP	Combination stabilized walking BP		
Octreotide sc	12.5–26 μg/kg	1 × 1 infusion 5–20 min before walking								
Protocol 6										
Octreotide sc	1.2–2.0 μg/kg	1 × 1	Open	8	MSA PAF	7–30 months	Duration of daily walking	No improvement of orthostatic tolerance		
**Ephedrine**										
Ephedrine	6–24 mg	TID	Single-center, placebo-controlled randomized, double-blind, blocked, crossover fashion	8	PAF MSA	2-day placebo run-in period (single-blind) Double-blind titration and maintenance period of 3–5 days each 4 days placebo wash out	Supine mean SBP, DBP, HR standing mean SBP, DBP, HR (after 1 min) ability to stand incidence of SH	Ephedrine did not significantly increase standing BP (*p* > 0.05) compared to placebo Midodrine significantly improved standing SBP and DBP compared to ephedrine (*p* < 0.05) and placebo (*p* < 0.001) Ephedrine (*p* < 0.01) and midodrine (*p* < 0.001) significantly increased supine BP compared to placebo without any difference between the two treatment arms Midodrine (*p* < 0.01) but not ephedrine led to an increased ability to stand	Ephedrine SH dizziness/lightheadedness photosensitivity disequilibrium	Fouad-Tarazi et al. (1995)
**Octreotide**										
DHE				28	PAF MSA DM SOH				Combination therapy (DHE and octreotide) finger cyanosis in MSA patient Octreotide nausea and abdominal cramps in DM pain due to injection impaired glucose tolerance loss of consciousness hyperpigmentation	Hoeldtke and Israel 1989
Protocol 1										
Octreotide sc	0.2 μg/kg 0.4 μg/kg	1 × 1	Single-center, placebo-controlled, randomized, crossover fashion Blinding unclear	28		1 day each treatment arm Unclear if conducted on consecutive days	Average semirecumbent BP	With exception of patients with SOH, BP significantly increased (*p* < 0.05)		
Protocol 2										
Octreotide sc	0.4 μg/kg 0.8 μg/kg 1.2 μg/kg 1.6 μg/kg	1 × 1 at the end of breakfast	Single-center, placebo-controlled, randomized, single-blind, crossover design	15	PAF MSA SOH	1 day each treatment arm Unclear if conducted on consecutive days	BP during walking walking time	In MSA and PAF octreotide significantly improved walking time *p* < 0.01 and upright BP *p* < 0.05 Treatment combination (DHE and octreotide) improved upright BP only partially		
DHE sc in combination with	12 μg/kg	1 × 1 60 min before breakfast								
Caffeine	250 mg	30 min before breakfast								
Protocol 3										
Octreotide sc	1.2 μg/kg 0.3 μg/kg 0.9 μg/kg	1 × 1 bolus priming dose continuous infusion over 70 min Administered 10 min before tilt table test	Single-center, placebo-controlled, randomized, crossover fashion Blinding unclear	17	PAF MSA DM	1 day each treatment arm Unclear if conducted on consecutive days	Upright mean BP (tilt table)	Continuous and bolus sc octreotide improved tilt table tolerance Octreotide infusion significantly (*p* < 0.05) increased BP		
Protocol 4										
Octreotide sc	1.2 μg/kg	1 × 1 10 min before tilting	Single-center, placebo-controlled, randomized, crossover fashion Blinding unclear	6	PAF MSA	1 day each treatment arm Unclear if conducted on consecutive days	Upright mean BP (tilt table)	Only combination therapy significantly (*p* < 0.05) improved OH		
DHE sc	10 μg/kg	1 × 1 70 min before tilting								
Combination										
Protocol 5										
DHE sc and propanolol	12–20 μg/kg 30 mg	1 × 1 60–90 min before octreotide	Open	3	SOH		Walking BP	Combination stabilized walking BP		
Octreotide sc	12.5–26 μg/kg	1 × 1 infusion 5–20 min before walking								
Protocol 6										
Octreotide sc	1.2–2.0 μg/kg	1 × 1	Open	8	MSA PAF	7–30 months	Duration of daily walking	No improvement of orthostatic tolerance		
Octreotide s.c.	100 μg	1 × 1	Single-center, randomized, placebo-controlled, double-blind, crossover fashion	9	MSA	1 day each treatment On 3 successive days	Standing BP standing HR NE levels	Tilt-test duration was significantly (*p* = 0.02) longer with octreotide compared to placebo or without treatment Minimal BP values did not differ during octreotide, placebo and control Octreotide significantly (*p* = 0.01) increased time until minimal BP was attained	SH	Bordet et al. (1995)

*AAN* autoimmune autonomic neuropathy, *BID* twice a day, *CGI* clinical global impression of change, *COMPASS-OD* Composite Autonomic Symptom Scale, *DBP* diastolic blood, *DAN* diabetic autonomic neuropathy, *DHE* Dihydroergotamine, *DL-DOPS* 3,4-DL-threo-dihydroxyphenylserine, *DM* diabetes mellitus, *h* hour, *E/I* ratio expiration: inspiration ratio, *HR* heart rate, *mg* milligram, *MSA* multiple system atrophy, *NE* norepinephrine, *NDAN* non-diabetic autonomic neuropathy (n) OH (neurogenic) orthostatic hypotension, *OHDAS* Orthostatic Hypotension Daily Activity Scale, *OHQ* Orthostatic Hypotension Questionnaire Q1 = dizziness, lightheadedness, feeling faint, or feeling like you might black out, *OHSA* Orthostatic Hypotension Symptom Assessment, *PAF* pure autonomic failure, *PD* Parkinson’s disease, *PRA* plasma renin activity, QD once a day (S)BP (systolic) blood pressure, *SH* supine hypertension, *sc* subcutaneous, *SOH* sympathotonic orthostatic hypotension, *TID* three times a day, *w* week, *μg* microgram

**Table 3:** Pharmacological interventions for treatment of post-prandial hypotension—available studies

Compound	Dosage	Schedule	Study design	Sample size	Patients	Duration	Outcome measures	Results	Safety issues	Study group
**Acarbose**										
Acarbose	100 mg	1 × 1 20 min before meal	Single-center, crossover fashion Phase 1: single-blind, placebo-controlled nonrandomized (*n* = 4) Phase 2 double-blind, placebo-controlled randomized (*n* = 9)	13	PAF PD	1 screening day 1 day each treatment arm Unclear if conducted on consecutive days	Primary supine postprandial SBP supine postprandial DBP supine postprandial HR every 5 min for 90 min after intervention Secondary glucose level insulin level pre-meal baseline, post-meal: 30, 45, 60 90 min Tertiary plasma catecholamine levels CO TPR FVR	Acarbose significantly (*p* < 0.01) reduced postprandial hypotension measured by SBP and DBP	No adverse effects	Shibao et al. (2007a)
Acarbose	50 mg	1 × 1 immediately before liquid meal	Single-center, randomized, double-blind, placebo- controlled, crossover fashion	15	DM type 2	1 day each treatment arm Unclear if conducted on consecutive days	Postprandial SBP postprandial DBP postprandial HR MAP MCA velocities Position of BP measurements unclear	Acarbose led to significant higher postprandial SBP and MAP (both *p* = 0.03) Significance was not reached for DBP (*p* = 0.07) and HR (*p* = 0.69)	Not mentioned	Madden et al. (2015)
**Octreotide**										
Octreotide sc	Protocol 1: 0.2 μg/kg 0.4 μg/kg Protocol 2: 0.1–0.6 μg/kg Protocol 3: 0.2 μg/kg 0.8 μg/kg	1 × 1 at breakfast	Single-center, placebo-controlled, randomized, crossover fashion Blinding unclear	8 Protocol 1: 6 Protocol 2: 7 Protocol 3: 4	Autonomic neuropathy alcoholism PD DM	1 day each treatment arm Unclear if conducted on consecutive days	Postprandial sitting mean BP (protocol 1) Postprandial upright BP (Protocol 2) Hormonal measurements (neurotensin, vasoactive intestinal peptide, substance P, human pancreatic polypeptide) (protocol 3)	Reduction of postprandial hypotension (*p* < 0.001) Octreotide increased upright BP during postprandial phase	Abdominal cramps and nausea in gastroparesis diabeticorum SH (BP 200/125 mmHg) Hyperglycemia	Hoeldtke et al. (1986a)
Octreotide sc	0.4 μg/kg	1 × 1 before 50-g glucose drink	Single-center, placebo-controlled, randomized, crossover fashion Blinding unclear	11	MSA PAF	1 day each treatment arm Conducted on consecutive days	Postprandial sitting mean BP plasma NE levels	Reduction of postprandial hypotension (*p* < 0.01)	Gastrointestinal side effects Hyperglycemia	Hoeldtke et al. (1989a)
Octreotide sc				16	PAF MSA DM CRF				Octreotide gastrointestinal side effects	Hoeldtke et al. (1998)
Protocol 1:										
Octreotide sc	0.5 μg/kg	1 × 1 15 min after begin of breakfast	Single-center, placebo-controlled, randomized, crossover fashion Combination therapy administered on last day No blinding	9		1 day each treatment arm Unclear if conducted on consecutive day	Postprandial sitting mean BP	Octreotide significantly increased mean postprandial BP (*p* < 0.01) with respect to placebo Midodrine reversed postprandial blood pressure drop only partially Combination accentuated the effect of octreotide	Midodrine pruritus of scalp urinary urgency SH	
Midodrine	5 mg	1 × 1 30 min before breakfast								
Protocol 2:										
Midodrine	5 mg 10 mg	1 × 1 30 min before breakfast	Single-center, placebo-controlled, randomized, crossover fashion Blinding unclear	10		1 day each treatment arm On consecutive days	Postprandial sitting mean BP	Midodrine significantly increased BP with respect to placebo (*p* < 0.01) 10 mg lead to significant higher BP increase than 5 mg of midodrine (*p* < 0.05)		
Protocol 3:										
Octreotide sc	1.0 μg/kg	1 × 1	Single-center, randomized, crossover fashion No placebo No blinding	12		1 day each treatment arm Unclear if conducted on consecutive days	Standing time	Octreotide significantly improved standing time (*p* = 0.0034) Midodrine did not differ from placebo (*p* = 0.11) Combination therapy was more effective than octreotide alone (*p* < 0.05)		
Midodrine	10 mg	1 × 1								
Combination		1 × 1								
**Caffeine**										
Caffeine				9	DM PAF alcoholism				Caffeine not mentioned	Hoeldtke et al. (1989b)
Protocol 2										
Caffeine	250 mg	1 × 1 30 min before breakfast	Single-center, randomized, placebo-controlled, crossover fashion No blinding	5		1 day each treatment arm On consecutive days	Postprandial sitting mean BP	Caffeine and DHE prevented postprandial OH only partially Combination therapy had a significant greater effect on postprandial hypotension (*p* < 0.01) and BP than monotherapy with either caffeine or DHE	DHE supine BP	
DHE sc	10μg/kg	1 × 1 60 min before breakfast								
Combination										
Protocol 1										
DHE sc	6.5μg/kg 13 μg/kg	1 × 1	Single-center, randomized, placebo-controlled, crossover fashion Blinding unclear	8		1 day each treatment arm On consecutive days	Supine mean BP upright mean BP (after 2 min) postprandial BP	Significant increase (*p* < 0.001) of upright BP and supine BP (*p* < 0.01) Average orthostatic BP drop was similar compared with placebo DHE failed to prevent postprandial hypotension		

*BP* blood pressure, *CO* cardiac output, *CRF* chronic renal failure, *DBP* diastolic blood, *DHE* Dihydroergotamine, *DM* diabetes mellitus, *FVR* Forearm vascular resistance, *HR* heart rate, *kg* kilogram, *MAP* mean arterial pressure, *MCA* middle cerebral arterial pressure, *mg* milligram, *min* minutes, *MSA* multiple system atrophy, *NE* norepinephrine, *PAF* pure autonomic failure, *PD* Parkinson’s disease, *SBP* systolic blood pressure, *sc* subcutaneously, *SH* supine hypertension, *TPR* total peripheral resistance, *μg* microgram

**Table 4:**
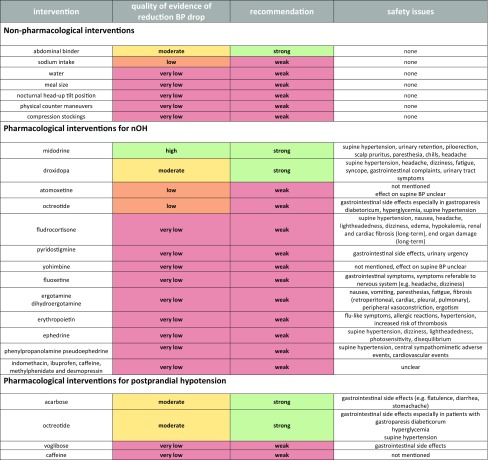
Quality of evidence (high, moderate, low, very low) and recommendations (strong, weak) for non-pharmacological and pharmacological treatment of nOH and post-prandial hypotension according to GRADE (Leone et al. 2013; https://gradepro.org)

Three main aspects penalize the quality of evidence for therapeutic interventions: first, studies are mostly small-sized and include populations of patients with nOH of different etiologies, which limits information on safety and efficacy in single diseases; second, for some non-pharmacological interventions (e.g., water, meal size, nocturnal head-up tilting, physical countermaneuvers) it is not possible to achieve a placebo-controlled study design; third, replication in larger placebo-controlled randomized clinical trials are often missing for those pharmacological measures, which showed positive results in pilot studies—such lack of data mirrors the challenges of running clinical trials in orphan diseases, where multi-center, adequately powered studies are hindered by limited commercial interest (Kaufmann et al. 2015).

## Approach to nOH and post-prandial hypotension in clinical practice

Cornerstone of therapy includes both non-pharmacological and pharmacological measures applied in a stepwise manner.

Non-pharmacological treatment options remain pivotal for the treatment of autonomic failure (see Table 5). With exception of abdominal binders (high quality of evidence, strong recommendation) the quality of evidence for all other non-pharmacological interventions is low to very low, and recommendation therefore weak. However, given the favorable safety profile of non-pharmacological interventions, patients should be advised and motivated to apply such strategies on a regular basis. Conservative interventions include adequate fluid (1.5–2 l/day) and salt (6–10 g/day) intake (Biaggioni 2014), as well as abdominal binders, alone or in combination with compression stockings to reduce venous pooling. Bolus ingestion of 500 ml water may diminish BP fall in the following 30 min, and can be therefore purposely applied by the patient based on planned activities. Patients should be advised to stand up slowly and to perform physical countermaneuvers (leg crossing, squat position, bending forward) in case of dizziness. Sleeping in a head-up tilt position should be recommended to prevent supine hypertension and reduce nocturnal pressure natriuresis (Fanciulli et al. 2016b). Patients should be also educated to avoid trigger factors such as alcohol, heat exposure and large carbohydrate- or fat-rich meals and be aware that Valsalva-like maneuvers (e.g., micturition and defecation) may precipitate hypotensive episodes.

**Table 5:** Non-pharmacological management of nOH

Increase in salt (6–8 g/day) intake
Increase in fluid (1.5–2 l/day) intake
Bolus ingestion of 500 ml water
Abdominal binders alone or with compression stockings
Physical countermaneuvers: leg crossing, squat position, bending forward
Sleeping with head-up tilt 30°
Avoidance of large fat- or carbohydrate-rich meals
Avoidance of trigger factors: alcohol, heat exposure, physical exertion
Awareness of Valsalva-like maneuvers (e.g., micturition, defecation)

When aforementioned measures have been implemented and do not provide satisfactory relief, pharmacological therapy is required in addition. Pharmacological options are summarized in Table 6. As a general rule, drug choice should be tailored on the basis of expected benefit, relevant comorbidities, and potential adverse events. In younger, mobile patients, one may treat nOH more vigorously to warrant autonomy and symptoms’ control in daily life. To the contrary, treatment of nOH may be less relevant than limiting polypharmacy in older, wheel-chair bound patients.

**Table 6:** Pharmacological management of nOH and post-prandial hypotension

*Orthostatic hypotension* (CAVE: last medication ≥ 4 h before bedtime)
Midodrine 2.5–10 mg; TID Fludrocortisone 0.1–0.2 mg; QD Droxidopa 3 × 100–600 mg; TID Atomoxetine 18 mg; QD
Ephedrine 25–50 mg; TID
Erythropoietin in combination with iron supplementation In selected cases: pyridostigmine, yohimbine, ergotamine, dihydroergotamine, ephedra alkaloids, desmopressin, indomethacin, fluoxetine
P*ost-prandial hypotension* (administer before meal)
Acarbose 100 mg
Octreotide 1 μg/kg sc (contraindication: DM)
Caffeine 250 mg

*DM* diabetes mellitus, *h* hours, *kg* kilogram, *mg* milligram, *μg* microgram, *QD* once a day, *TID* three times a day

At present only midodrine and droxidopa (the latter in the US and Japan only) have been approved for treatment of nOH, while the remaining substances are prescribed in an off-label regimen.

Based on available literature, the quality of evidence is high and recommendation level is strong for midodrine. Starting dose is 2.5 mg two to three times a day, which can be increased up to 10 mg 3 times a day. Midodrine can be administered in mono- or combined therapy with plasma volume expanders, such as fludrocortisone (Lahrmann et al. 2006). Use of midodrine is contraindicated in patients with severe cardiac disease, renal failure and urinary retention (McClellan et al. 1998).

Quality of evidence is moderate for droxidopa and the level of recommendation strong. Droxidopa should be initiated with a dosage of 100 mg three times a day and could be titrated up to a maximum dosage of 600 mg three times a day (Ricci et al. 2015). Notably, long-term data on safety and efficacy of droxidopa are still missing, limiting its use as first line therapy in clinical practice.

Fludrocortisone (quality of evidence very low, weak recommendation strength) can be considered as second-line agent, either as monotherapy (0.1–0.2 mg daily) or in combination with midodrine, if supine hypertension has been excluded (Freeman 2008). Importantly, fludrocortisone is contraindicated in patients with heart failure, kidney failure and hypertension (Ricci et al. 2015) and electrolyte monitoring is recommended to rule out hypokalemia.

Atomoxetine (18 mg, single dose) showed promising results in recent studies and may be considered in selected patients with refractory nOH, proven they have no cerebrovascular or cardiac comorbidities and are not on treatment with monoamine oxidase inhibitors. Data on long-term efficacy and safety are missing for atomoxetine, determining a low quality of evidence and a weak recommendation level.

Quality of evidence is very low and recommendation weak for ephedrine (25–50 mg three times a day) (Freeman 2008). Erythropoietin in combination with iron supplementation may have a positive impact on symptoms of nOH, especially in anemic patients, but risk of supine hypertension and polycythemia have to be considered (Kaufmann et al. 2015; Freeman 2008). For pyridostigmine, yohimbine, ergotamine, dihydroergotamine, ephedra alkaloids, desmopressin, indomethacin and fluoxetine the quality of evidence is very low, for octreotide low. Based on inconsistent results implications for clinical practice for all these treatment options remain investigational and therefore, recommendations are weak. Considering individual risk–benefit ratio, abovementioned supplementary agents might be evaluated in selected cases.

It has to be emphasized that, although anti-hypotensive agents are frequently prescribed in combination in clinical practice, there is little evidence on this approach, especially regarding long-term benefits and safety.

Hypertensive BP fluctuations in the supine position are a common problem in patients with cardiovascular autonomic failure and limit management of nOH. Patients taking any anti-hypotensive medication should be educated to avoid the recumbent position during daytime (Friedrich et al. 2010) and sleep with a head-up position overnight (Ten Harkel et al. 1992). All anti-hypotensive agents should not be taken < 4 h before bedtime and BP in the supine position should be monitored regularly.

In severe postprandial hypotension with limited response to conservative management (avoidance of large fat- or carbohydrate-rich meals and alcohol, increase in fluid and salt intake), acarbose in a dosage of 100 mg can be used in selected cases (quality of evidence is moderate, recommendation strong). Alternatively, subcutaneous octreotide (quality of evidence moderate, recommendation strong, 1 μg/kg) may be helpful (Wenning et al. 2005). Octreotide is contraindicated in patients with diabetes mellitus, given the risk of causing post-prandial hyperglycemia.

Although the quality of evidence is low and recommendation weak, caffeine in a dosage of 250 mg may be considered as further alternative, given its favorable safety profile.

## Conclusion

The target of nOH management is improvement of patient’s quality of life by warranting mobility and preventing injurious falls due to syncope and pre-syncope. Although nOH has a high prevalence in the aging population, evidence-based data are limited. The mainstay of clinical management is, therefore, an individually tailored therapy, based on both non-pharmacological and pharmacological measures, which needs to be regularly evaluated and adapted over time, especially if the case of dynamic clinical scenarios, like in neurodegenerative diseases.
